# Deep learning for freezing of gait assessment using inertial measurement units: a multicentre validation study

**DOI:** 10.1038/s41531-026-01407-7

**Published:** 2026-06-02

**Authors:** Po-Kai Yang, Juha Carlon, Maaike Goris, Emilie Klaver, Jorik Nonnekes, Richard J. A. van Wezel, Lisa Alcock, Alison J. Yarnall, Lynn Rochester, Clint Hansen, Christian Schlenstedt, Walter Maetzler, David Buzaglo, Marina Brozgol, Jeffrey M. Hausdorff, Alice Nieuwboer, Moran Gilat, Pieter Ginis, Bart Vanrumste, Benjamin Filtjens

**Affiliations:** 1 KU Leuven, Department of Electrical Engineering (ESAT), Leuven, Belgium; 2 KU Leuven, Department of Rehabilitation Sciences, Neurorehabilitation Research Group (eNRGy), Leuven, Belgium; 3 Medical Spectrum Twente, Department of Neurology and Clinical Neurophysiology, Enschede, The Netherlands; 4 Radboud University Medical Centre, Donders Institute for Brain, Cognition and Behaviour, Department of Rehabilitation, Centre of Expertise for Parkinson and Movement Disorders, Nijmegen, The Netherlands; 5 Radboud University, Donders Institute for Brain, Cognition and Behaviour, Department of Neurobiology, Nijmegen, The Netherlands; 6 University of Twente, MedTech Centre, Department of Biomedical Signals and Systems, Enschede, The Netherlands; 7 Newcastle University, Translational and Clinical Research Institute, National Institute for Health and Care Research (NIHR) Newcastle Biomedical Research Centre (BRC), Faculty of Medical Sciences, Newcastle Upon Tyne, UK; 8 Newcastle Upon Tyne Hospitals NHS Foundation Trust, Newcastle Upon Tyne, UK; 9 University Medical Center Schleswig-Holstein Campus Kiel, Department of Neurology, Kiel, Germany; 10 Medical School Hamburg, Institute of Interdisciplinary Exercise Science and Sports Medicine, Hamburg, Germany; 11 Tel Aviv Sourasky Medical Center, Center for the Study of Movement, Cognition and Mobility, Neurological Institute, Tel Aviv, Israel; 12 Tel Aviv University, Department of Physical Therapy, Gray Faculty of Medical and Health Sciences and Sagol School of Neuroscience, Tel Aviv, Israel; 13 Rush University Medical Center, Rush Alzheimer’s Disease Center and Department of Orthopaedic Surgery, Chicago, IL USA; 14 Clario, part of Thermo Fisher Scientific, Leuven, Belgium; 15 Delft University of Technology, Department of Engineering Systems and Services (ESS), Delft, The Netherlands; 16 Delft University of Technology, Institute for Health Systems Science, Delft, The Netherlands

**Keywords:** Health care, Medical research, Neurology, Neuroscience

## Abstract

Video annotation is the gold standard for assessing Freezing of Gait (FOG) in Parkinsonian disorders, but it is time-consuming. Deep learning (DL)-based assessment of FOG using inertial measurement units ameliorates this problem but poses challenges. Particularly, the large heterogeneity between patients and assessment methods potentially affects detection performance between independent cohorts. To evaluate heterogeneity effects, we developed a DL model on a local cohort (85 participants; 2043 trials) and validated it across six external cohorts (256 participants; 1058 trials). Model-expert agreement on the percentage-of-time-frozen was strong locally (ICC = 0.886 [0.79, 0.90]) but reduced in external cohorts (ICC = 0.562 ± 0.141). Fine-tuning the DL model with just 50 min of external cohort data improved the ICC to 0.732 ± 0.138, approaching the lower boundary of the inter-rater agreement between two clinical raters using video annotation (ICC = 0.73–0.99). Therefore, while unified standards are still being developed, we propose a human-in-the-loop workflow as an effective intermediary and present a proof-of-concept web-based platform for fine-tuning and expert review (aidfog.be).

## Introduction

Freezing of gait (FOG) is a debilitating motor symptom observed predominantly in people with Parkinson’s disease (PD) and related disorders^1,2^. FOG is currently clinically defined as a “brief, episodic absence or marked reduction of forward progression of the feet despite the intention to walk and reach a destination”^3^. FOG significantly compromises mobility, independence, and quality of life and increases the risk of falls^4–6^. It is estimated that FOG affects up to 80% of people over the course of PD^7,8^, highlighting its substantial prevalence and clinical relevance.

**Fig. 1:**
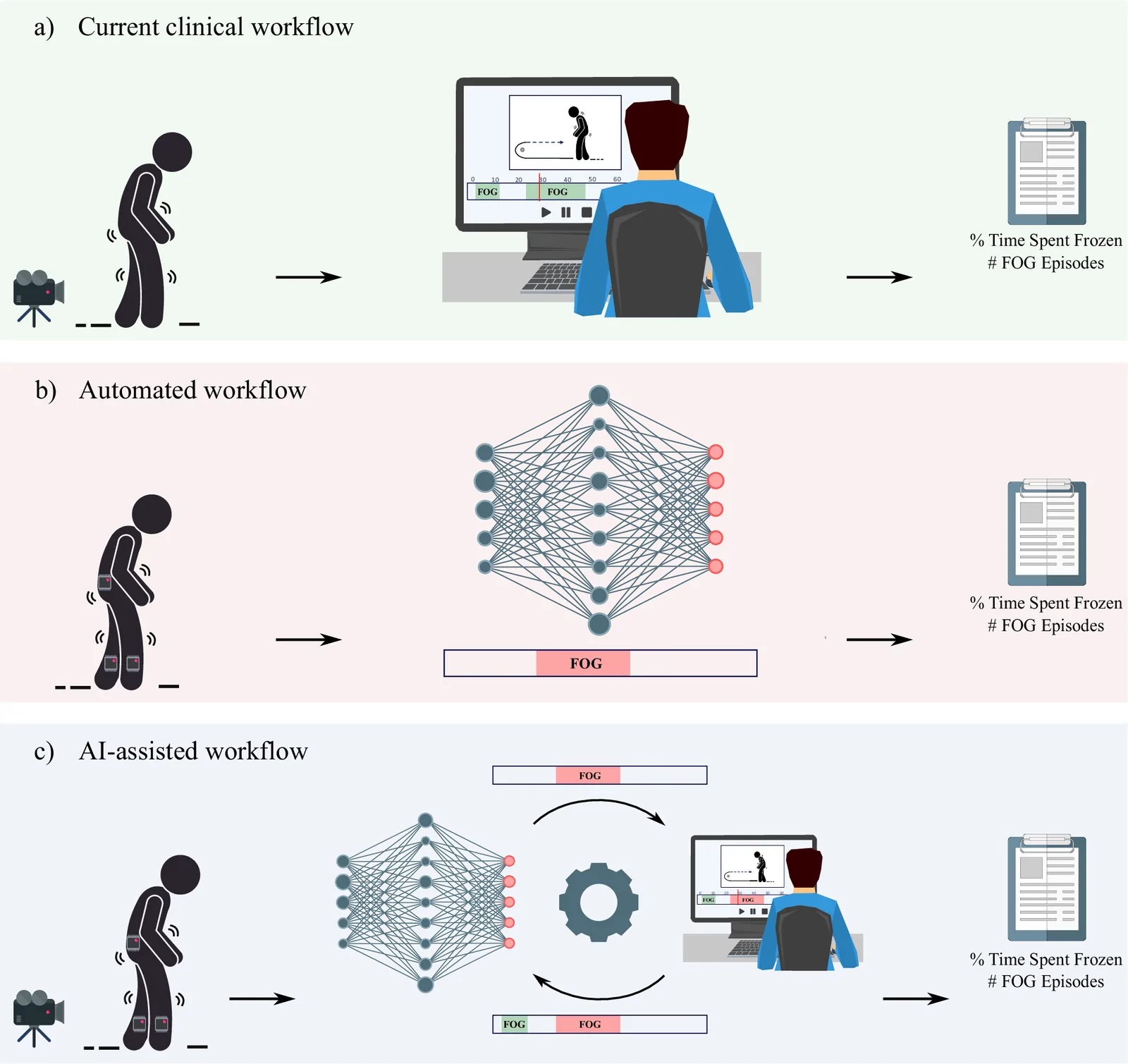
Three workflows for freezing of gait assessment in Parkinson’s disease. The figure compares the current clinical workflow against automated and AI-assisted alternatives. **a** Current clinical workflow: a trained human evaluator reviews camera footage of gait tasks and manually identifies freezing of gait (FOG) episodes on a frame-by-frame basis. **b** Automated workflow: gait data collected from inertial measurement units (IMUs) are processed by a deep-learning algorithm (DLA), which automatically identifies potential FOG episodes without human intervention. **c** AI-assisted “human-in-the-loop” workflow: the DLA provides an initial evaluation of the gait task using IMU data; a human evaluator then reviews and refines the annotations, incorporating additional information from camera footage, and the refined predictions can subsequently be used to fine-tune the DLA. In all three workflows, FOG severity is quantified using outcome measures derived from the identified FOG episodes, namely the percentage of time spent frozen (%TF) and the number of FOG episodes (#FOG). FOG freezing of gait, IMU inertial measurement unit, DLA deep-learning algorithm, %TF percentage of time spent frozen, #FOG number of FOG episodes.

The gold standard to assess the presence and severity of FOG involves dedicated gait tasks, so-called FOG-provoking tasks, that challenge dynamic motor-cognitive or motor-sensory control by including triggers that frequently elicit FOG (e.g., gait initiation, narrow passageways, cognitive dual tasks, and rapid 180^∘^ or 360^∘^ turning)^9–13^. Camera recordings capture these gait tasks, which are then meticulously reviewed by trained evaluators to annotate individual FOG episodes (Fig. 1a)^14^. This method allows FOG severity to be quantified as the percentage of time spent with FOG compared to the total task duration (%TF)^15^, and the number of FOG episodes (#FOG). However, this process is extremely labor-intensive, requiring trained evaluators to manually review video footage on a frame-by-frame basis, which slows the overall assessment pace. Additionally, human judgment introduces variability across evaluators^16^, which complicates the standardization of FOG assessment and impacts the reliability of determining the treatment response^11,17–19^.

Recent advances in machine learning, particularly driven by deep learning (DL), combined with the ubiquity and affordability of wearable sensors such as inertial measurement units (IMUs), hold promise to overcome the limitations of manual FOG annotation (Fig. 1b). Prior research has highlighted that DL algorithms (DLA), when combined with IMUs, enable reliable assessment of FOG^11,20^. Nevertheless, the reliability of these automated analyses was constrained by the possible pitfall of overfitting the model to detect such a complex and heterogeneous symptom when only limited sample sizes are available or diminished reproducibility stemming from the absence of multicentre datasets^20,21^. Consequently, DLAs trained on a specific dataset may not generalize well to detect FOG in other datasets. In this field, poor generalization between datasets is driven by three key sources of variability^17^: (1) variability in FOG-provoking tasks and protocols, where differences in task design and triggers elicit different movement patterns; (2) variability in annotation criteria and operational definitions, as the current FOG definition leaves room for subjective interpretation; and (3) variations in IMU configurations, including differences in sensor numbers and placements. These three categories are also the focus of current harmonization activities by the International Consortium for FOG (ICFOG) (https://icfog.org/.)^17,22^. Specifically, ICFOG is coordinating standardization across three working groups: (a) a definition working group with the objective to operationalize a consensus definition of FOG^19^; (b) a protocol working group evaluating the psychometric properties of a consensus-based FOG-provoking assessment battery (the “Giladi Protocol”; NCT06519279)^23^; and (c) a technology working group aiming to align and standardize the measurement technology used in clinic and research settings to progressively enable more efficient and automated FOG evaluation^20^.

Importantly, even if definition, protocol, and technology become fully standardized, variability will still remain due to factors that are inherent to patient spectrum differences across cohorts and recruitment pathways, and the residual subjectivity inherent to expert labeling. For this reason, our study explicitly intended to evaluate how heterogeneity impacts model generalizability across cohorts. Specifically, we define generalizability as the ability of a model trained on one cohort to maintain reliable performance across external cohorts with differing protocols, sensor setups, and patient spectra. Moreover, integration of DLAs into a clinical workflow could face obstacles due to the limited interpretability of the models, often referred to as the “black box” phenomenon^24^. One possible solution is a human-in-the-loop approach, in which clinicians review the model’s predictions and the rationale behind them (e.g., by determining which data patterns influenced a decision) before using those predictions to guide treatment, thereby building trust and transparency^25,26^.

To address these challenges and build on our earlier IMU-based FOG detection study^27^, we proceeded in three steps. First, we evaluated three state-of-the-art sequence learning architectures, including Multi-Stage Temporal Convolutional Network (MS-TCN)^28^, the Action Segmentation Transformer (ASFormer)^29^, and the Long-Term Context Action Segmentation model (LTContext)^30^. These architectures were benchmarked to identify the best model for FOG detection across external datasets and quantify how cohort differences affect model performance. Next, to adapt the model to possible cohort differences, we compared an adaptation strategy on the external datasets, i.e., by fine-tuning the model with varying amounts of annotated data, and comparing this with retraining a model from scratch. Finally, to encompass model fine-tuning and support a “human-in-the-loop” approach, we introduced an AI-assisted workflow (Fig. 1c), where the DLA supports but does not replace researchers in annotating FOG episodes. To operationalize this workflow and facilitate future user studies, we built the AID-FOG (AI-Driven Freezing of Gait) platform, a proof-of-concept web application that could serve two purposes: (1) enhancing transparency and trust by enabling researchers to review and refine model predictions; and (2) fine-tuning the DLA using these refined predictions, thereby improving its generalization to data collected from different cohorts. For a detailed design process of the platform and a live demo, refer to the Supplementary Information (Supplementary Figs. 6 and 7) and Supplementary Video 1.

## Results

### Dataset and participant characteristics

We included datasets from seven cohorts collected across eight universities. The local cohort comprised four datasets from various KU Leuven projects, including those used in previous studies^27,31,32^, as well as data from an ongoing study (ClinicalTrials.gov NCT05511597). These were collectively used as the local training dataset. The six external cohorts included: (1) a non-public dataset from Radboud University^33^, (2) a public dataset from Stanford University^34^, (3) a public dataset from the Federal University of ABC^35^, (4) a public dataset from the University of Twente^36,37^, (5) a public dataset from Beijing Xuanwu Hospital^38,39^, and (6) a non-public multicenter dataset from the Mobilise-D project^13^, which includes data from KU Leuven, University Medical Center Schleswig-Holstein Campus Kiel, and Tel Aviv Sourasky Medical Center. As outlined in the Introduction, these cohorts collectively capture the three key sources of variability: the Stanford and Radboud cohorts employed different FOG-provoking tasks; the Federal, Twente, and Xuanwu cohorts introduced variations in the number of IMU sensors; and annotation criteria varied slightly across cohorts as detailed in section “Methods.” Additionally, the Mobilise-D cohort reflected patient spectrum differences that, while not amenable to standardization, represent a persistent source of domain shift.

Table 1 summarizes the patient and data characteristics across the seven cohorts. All participants had a clinical diagnosis of idiopathic PD. Participants varied in age and disease duration, with the Stanford cohort having the youngest average age (58 ± 5 years) and the Radboud cohort the oldest (72 ± 4 years). Among the external cohorts, Radboud and Stanford featured distinct FOG-provoking tasks, e.g., 15-meter walking with volitional stopping (Radboud) and slalom with figure-eight turns (Stanford), which differed from those used in the KU Leuven cohort, such as the TUG, 360^∘^ turning, 5-meter walk, and slalom tasks. Additionally, while most datasets used five IMUs, fewer IMUs were available for the Federal, Twente, and Xuanwu cohorts. As described in the section “Data harmonization,” as the original Stanford and Twente publications only specified “ankle-mounted” and “foot-mounted” sensors without naming the exact bones, we harmonized placements of ankle-mounted IMUs to correspond to tibia positions and their foot-mounted IMUs to talus positions. Based on the New Freezing of Gait Questionnaire (NFOG-Q) and the Movement Disorders Society Unified Parkinson’s Disease Rating Scale (MDS-UPDRS) III scores, the Radboud, Federal, Twente, and Xuanwu cohorts exhibited similar disease and FOG severity to KU Leuven, while Mobilise-D had lower scores, indicating milder disease severity and lower probability for FOG^40^. Indeed, the average FOG episode duration in Mobilise-D was the shortest among all datasets and only 0.983 s, suggesting a predominance of mild freezers experiencing very short FOG episodes.

**Table 1:** Dataset characteristics

	KU Leuven	Radboud	Stanford	Federal	Mobilise-D	Twente	Xuanwu
**Data characteristics**							
Number of patients	85	25	7	35	156	21	12
Number of freezers	54	18	7	26	98	21	10
Number of trials	2043	694	60	71	156	21	56
Dataset duration (min)	845.15	435.52	85.64	142.00	156.20	920.00	169
Number of FOG episodes	1798	280	211	173	381	1006	325
Mean/Median FOG episode duration (s)	3.19/1.31 ± 6.20	7.62/5.00 ± 9.64	6.24/4.00 ± 10.31	9.32/2.94 ± 19.28	0.98/0.59 ± 1.29	13.33/6.00 ± 17.01	13.75/8.00 ± 14.80
Total FOG duration (min)	95.69	35.57	21.95	26.87	6.24	223.54	74.48
FOG-provoking tasks	TUG/360^∘^ turns (w/wo stops), 5m walk, slalom	15 m walk with stopping, turns	Slalom with ellipses and figure-eights	360^∘^ turns	360^∘^ turns	Walking/turning	Walking/turning
Medication states	ON+OFF	OFF	OFF	ON	ON	OFF	OFF
IMU sensor positions	Pelvis + Lower legs (L/R) + Feet (L/R)	Full-body (17)	Chest, Lumbar, Ankles/Feet (L/R)	Left foot	Pelvis + Lower legs (L/R) + Feet (L/R)	Right ankle	Lower legs (L/R)
IMUs used /total	5 / 5	5 / 17	5 / 7	1 / 1	5 / 5	1 / 1	2/2
**Participant characteristics**							
Age (years)	67 ± 8	72 ± 4	58 ± 5	65 ± 10	63 ± 9	71 ± 8	69 ± 8
Gender (M:F)	62:23	19:6	4:3	19:16	101:55	17:4	6:6
PD duration (years)	10 ± 5	10 ± 5	10 ± 2	8 ± 4	8 ± 5	10 ± 5	9 ± 7
FOGQ	-	-	-	-	-	-	16.20 ± 4.2
NFOG-Q	20.36 ± 9.27	18.12 ± 5.35	-	17.72 ± 5.63	13.38 ± 6.06	16.30 ± 5.00	-
MDS-UPDRS III (OFF)	-	36.36 ± 13.88	-	-	-	53.90 ± 15.80	45.00 ± 16.00
MDS-UPDRS III (ON)	33.34 ± 12.24	-	-	31.83 ± 14.99	28.66 ± 12.71	41.10 ± 23.40	-
MoCA	26.36 ± 2.62	-	-	18.55 ± 7.23	26.49 ± 2.67	24.00 ± 4.00	23.60 ± 3.60
MMSE	27.93 ± 3.51	27.32 ± 2.66	-	26.63 ± 2.72	-	-	28.20 ± 1.50
HY Scale (mean/median ± STD)	2.35/2 ± 0.53	2.24/2 ± 0.44	-	2.91/3 ± 0.51	1.88/2 ± 0.53	1.00/0.5 ± 1.10	-

*FOG* freezing of Gait, PD Parkinson’s disease, *FOGQ* Freezing of Gait Questionnaire, *NFOG-Q* New Freezing of Gait Questionnaire, MDS-UPDRS Movement Disorders Society Unified Parkinson’s Disease Rating Scale, *MMSE* Mini-Mental State Examination, MoCA Montreal Cognitive Assessment.

MDS-UPDRS III scores were collected under OFF medication in the Radboud and Xuanwu datasets. For KU Leuven, OFF scores were available for participants from ref. ^32^, while the remaining participants were assessed under ON medication (only ON scores are reported here). The Federal and Mobilise-D cohorts provided ON scores only, whereas the Twente cohort provided scores under both conditions.

For model pre-training, 2043 gait trials from 85 participants were included in the KU Leuven cohort. The %TF was 11.32% (95.69 out of 845.15 min), with a total of 1798 FOG episodes observed. For external validation, 1058 trials from 256 participants across the six external cohorts were used. The %TF was 20.37% (388.65 out of 1908.36 min), with a total of 2376 FOG episodes recorded.

### Model development

We compared three state-of-the-art sequence learning architectures for FOG detection: MS-TCN, ASFormer, and LTContext. We first searched for optimal hyperparameters for each model using the internal KU Leuven dataset; the full results and selected hyperparameters are detailed in the Supplementary Information. In the second stage, each model was trained on the KU Leuven dataset with its optimal hyperparameters, and inference was performed on the external cohorts. The mean performance across external cohorts is summarized in Table 2, with per-dataset results reported in the Supplementary Information (Supplementary Table 6). LTContext was selected for further experiments, as it achieved the highest ICC for both %TF and #FOG across all external datasets. However, these ICC values still reflect poor generalization, which we further investigate in the section “Pretrained model externally validated.”

**Table 2:** Mean ICC for the evaluated models applied to the external datasets

	Mean value	
Model	ICC(%TF)	ICC(#FOG)
LTContext	**0.562 ±** **0.141**	**0.298 ±** **0.270**
ASFormer	0.494 ± 0.261	0.133 ± 0.138
MS-TCN	0.438 ± 0.232	0.179 ± 0.193

Bold values indicate the best result per column.

### Pretrained model externally validated

In the local KU Leuven cohort, the developed model achieved strong agreement with experts on %TF (ICC = 0.886, 95% confidence interval (CI) [0.79, 0.90]) and fair agreement on #FOG (ICC = 0.573, 95% CI [0.28, 0.71]). In trials with at least one FOG episode, it achieved a sample F1 of 0.291 and a segment F1@50 of 0.123. In non-FOG trials, it made 0.302 false positive (FP) episodes per trial and a mean FP duration per trial of 0.230 s, which helped maintain the ICC. These relatively low F1 scores, calculated at a strict, trial-wise level (see section “Metrics and statistics” for details), underscore the challenges of detecting FOG, even on local data. For a broader set of performance metrics (e.g., accuracy, recall, specificity, and precision) computed by pooling all participants within the KU Leuven dataset, please refer to Supplementary Table 7.

When tested on the external cohorts (Table 3), agreement in terms of FOG severity dropped: mean ICC for %TF across external datasets reduced to 0.562 ± 0.141 (mean ± standard deviation (SD) across six datasets; 95% CI [-0.01, 0.78]) and for #FOG to 0.298 ± 0.270 (mean ± SD across six datasets; 95 % CI [-0.16, 0.49]). The sample F1 across participants in FOG trials increased slightly to 0.390 ± 0.162 (mean ± SD across participants; F1@50 = 0.185 ± 0.113), but the #FP in non-FOG trials increased to 1.690 ± 1.579 per trial (mean ± SD across participants; mean FP duration 5.076 ± 5.939 s). This reduction reflects a lowered agreement in both %TF and #FOG, showing that differences between cohorts can reduce model performance. Notably, the higher F1 on external cohorts compared to the KU Leuven cohort is driven by the higher FOG prevalence in external cohorts (%TF = 20.37% vs. 11.32%) rather than better model performance, as confirmed by the concurrent increase in #FP per trial and the drop in ICC(%TF) from 0.886 to 0.562.

**Table 3:** Performance of DLA models trained from scratch, pre-trained, and fine-tuned

Method	FOG Trials		Non-FOG Trials		All Trials	
	F1	F1@50	#FPs/trial	FP duration/trial	ICC(%TF)	ICC(#FOG)
**Stanford**						
*Scratch (50 min)*	0.400	0.208	0.542	2.298	0.932	0.539
*Pre-trained*	0.240	0.082	0.125	0.125	0.616	0.556
→ *Tuned (50 min)*	0.331 (+37.9%)	0.149 (+81.7%)	0.404 (+223.2%)	1.537 (+1129.6%)	0.847 (+37.5%)	0.480 (−13.7%)
**Mobilise-D**						
*Scratch (50 min)*	0.170	0.056	2.375	1.507	0.337	0.187
*Pre-trained*	0.140	0.066	0.339	0.163	0.426	0.291
→ *Tuned (50 min)*	0.148 (+5.7%)	0.068 (+3.0%)	1.071 (+215.9%)	0.421 (+158.3%)	0.478 (+12.2%)	0.418 (+43.6%)
**Federal**						
*Scratch (50 min)*	0.297	0.079	0.528	1.235	0.676	0.105
*Pre-trained*	0.377	0.099	4.787	17.355	0.417	0.001
→ *Tuned (50 min)*	0.340 (−9.8%)	0.083 (−16.2%)	1.167 (−75.6%)	0.935 (−94.6%)	0.625 (+49.9%)	0.385 (+38400.0%)
**Radboud**						
*Scratch (50 min)*	0.213	0.136	0.240	0.754	0.444	0.189
*Pre-trained*	0.457	0.236	1.375	1.688	0.481	0.098
→ *Tuned (50 min)*	0.369 (−19.2%)	0.207 (−12.0%)	0.170 (−87.6%)	0.363 (−78.5%)	0.761 (+58.2%)	0.655 (+568.4%)
→ *FP removal*	0.457	0.236	0.000	0.000	0.893	0.642
**Twente**						
*Scratch (50 min)*	0.546	0.389	0.296	1.686	0.961	0.825
*Pre-trained*	0.501	0.249	1.052	5.932	0.610	0.095
→ *Tuned (50 min)*	0.435 (−13.2%)	0.260 (+4.4%)	0.204 (−80.6%)	0.951 (−83.9%)	0.838 (+37.4%)	0.877 (+822.1%)
**Xuanwu**						
*Scratch (50 min)*	0.440	0.350	0.700	1.975	0.522	0.596
*Pre-trained*	0.626	0.380	2.462	5.196	0.824	0.748
→ *Tuned (50 min)*	0.570 (-8.9%)	0.395 (+3.9%)	0.283 (-87.4%)	0.642 (-87.6%)	0.845 (+2.5%)	0.923 (+23.4%)
**Averaged**						
Scratch (50 min)	0.344 ± 0.131	0.203 ± 0.128	0.780 ± 0.730	1.576 ± 0.498	0.645 ± 0.236	0.407 ± 0.263
Pre-trained	0.390 ± 0.162	0.185 ± 0.113	1.690 ± 1.579	5.076 ± 5.939	0.562 ± 0.141	0.298 ± 0.270
→ Tuned (50 min)	0.366 ± 0.126 (−6.3%)	0.194 ± 0.112 (+4.5 %)	0.550 ± 0.410 (−67.5%)	0.808 ± 0.397 (−84.1%)	0.732 ± 0.138 (+30.2 %)	0.623 ± 0.214 (+108.9 %)

Results are reported for each dataset using 50 min annotated data for fine-tuning and training-from-scratch. Overall performance is summarized as the arithmetic mean and standard deviation across datasets. Percentage improvements are calculated relative to the pre-trained model using: $\frac{\,{\rm{Fine-tuned}}-{\rm{Pre-trained}}}{{\rm{Pre-trained}}}\times 100 \%$.

### Fine-tuned model externally validated

**Fig. 2:**
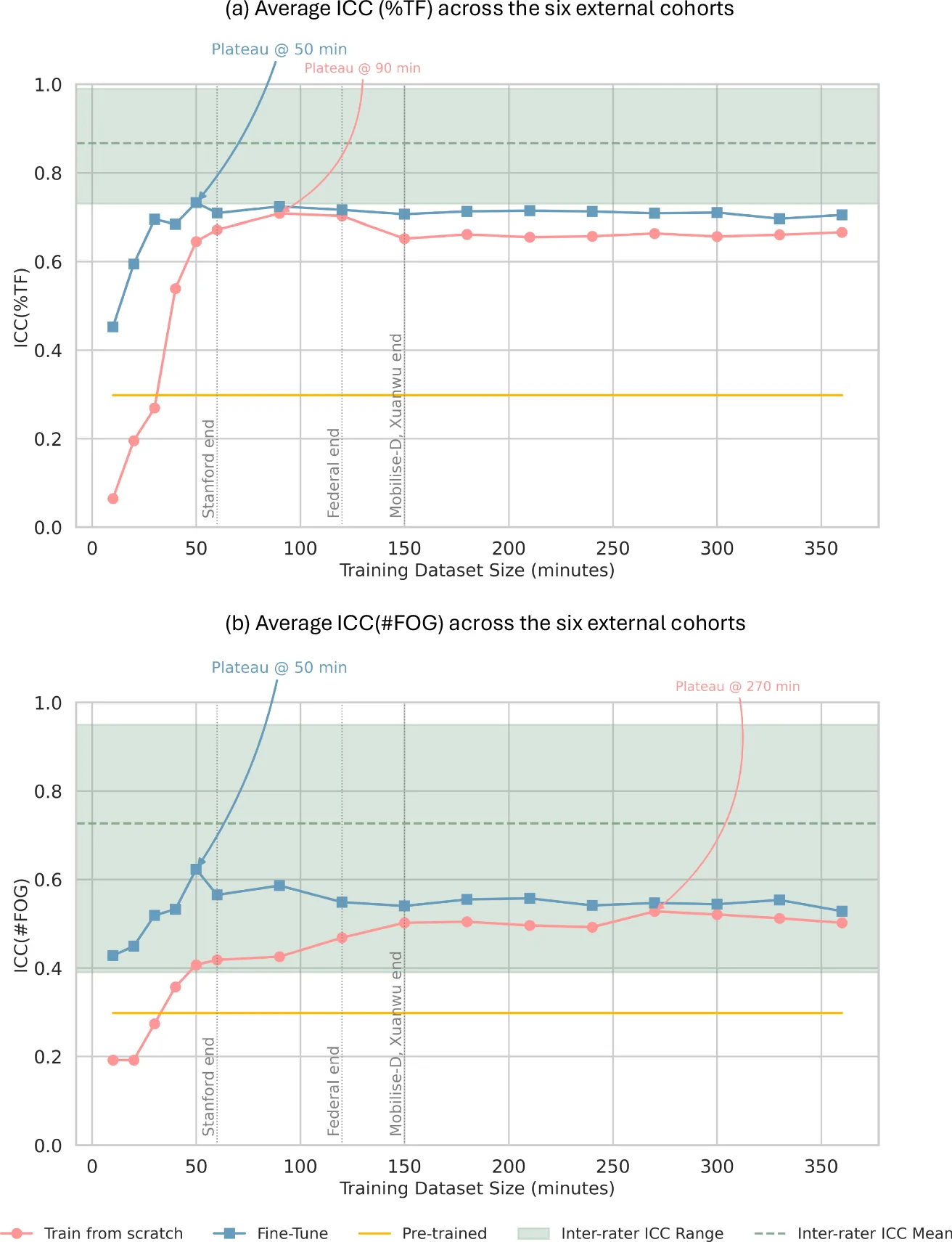
Effect of training data quantity on model agreement with expert annotations across six external cohorts. The figure shows two panels comparing three training strategies as a function of training dataset size. **a** Average intraclass correlation coefficient for the percentage of time spent frozen (ICC(%TF)) across the six external cohorts. **b** Average ICC for the number of FOG episodes (ICC(#FOG)) across the same cohorts. In both panels, the red dashed line with circle markers represents models trained from scratch on each external cohort; the blue solid line with square markers represents the KU Leuven-pretrained model fine-tuned on each external cohort; and the yellow solid horizontal line represents the KU Leuven-pretrained model evaluated without adaptation. Vertical gray lines indicate the training duration beyond which data were no longer available for a given cohort (labeled as “Stanford end'', “Federal end'', “Mobilise-D, Xuanwu end''). When a cohort lacked data at a specific time point, its last available value was carried forward to compute the cross-cohort average, based on the common assumption that model performance generally improves, or at least does not deteriorate, with more training data^65^. The green shaded area represents the range (minimum to maximum) of inter-rater ICCs for %TF and #FOG reported in previous studies^10,15,34,41 --43^, with the green dashed horizontal line indicating the reported mean. Fine-tuning raises ICC(#FOG) into the inter-rater range (0.390–0.950), while ICC(%TF) remains slightly below the lower bound of the inter-rater range (0.730-0.990), primarily due to limited improvement in the Mobilise-D cohort, which predominantly includes mild freezers. ICC intraclass correlation coefficient, %TF percentage of time spent frozen; #FOG number of FOG episodes.

To address the performance drop observed in the external datasets, we explored fine-tuning as an adaptation strategy for the LTContext model pre-trained on the local KU Leuven cohort and compared it against training a new model from scratch with randomly initialized weights directly on the external datasets. As shown in Fig. 2, fine-tuning the model with increasing amounts of data (from 10 to 360 min) consistently improved ICC scores for both %TF and #FOG compared to the pre-trained baseline model, with gains plateauing at around 50 min of training data. At that point, fine-tuned ICC(%TF) reached 0.732 ± 0.138 (+30.2 %), and ICC(#FOG) reached 0.623 ± 0.214 (+108.9 %). Models trained from scratch followed the same training trajectory when more annotated data was added but plateaued later at ICC(%TF) = 0.708 ± 0.195 after 90 min and ICC(#FOG) = 0.528 ± 0.244 after 270 min. This result underscores that fine-tuning the pre-trained model is more data-efficient for adapting to external cohorts than training a new model from scratch.

Nevertheless, as shown in Fig. 2, even after fine-tuning or training from scratch, ICC(%TF) remained at the lower boundary of the ICC ranges between human raters reported in the literature (0.730-0.990 for %TF and 0.390-0.950 for #FOG). Among all cohorts, Mobilise-D showed the smallest gains, likely due to the difficulty of annotating shorter FOG episodes during 360^∘^ turning-in-place tasks in mild freezers (see cohort-difference analysis below).

Our per-dataset analysis showed that fine-tuning mitigated cohort differences. On average, for challenge 1 (task differences), ICC(%TF) improved from 0.549 to 0.870 (+58.5%) and ICC(#FOG) from 0.327 to 0.568 (+73.7%). For challenge 2 (annotation criteria), ICC(%TF) increased from 0.616 to 0.847 (+37.5%), though ICC(#FOG) slightly declined from 0.556 to 0.480 (−13.7%). For challenge 3 (IMU variations), ICC(%TF) improved from 0.617 to 0.769 (+24.6%) and ICC(#FOG) from 0.281 to 0.728 (+159.1%). For challenge 4 (patient spectrum bias), ICC(%TF) increased from 0.426 to 0.478 (+12.2%) and ICC(#FOG) from 0.291 to 0.418 (+43.6%). Here, we report ICCs to support clinical interpretation. In the Supplementary Information (Supplementary Figs. 4 and 5), we demonstrate how fine-tuning on each dataset changes sample- and segment-level F1, mean #FP per trial, and mean FP duration per trial, and how these metric changes drive the observed ICC values.

**Fig. 3:**
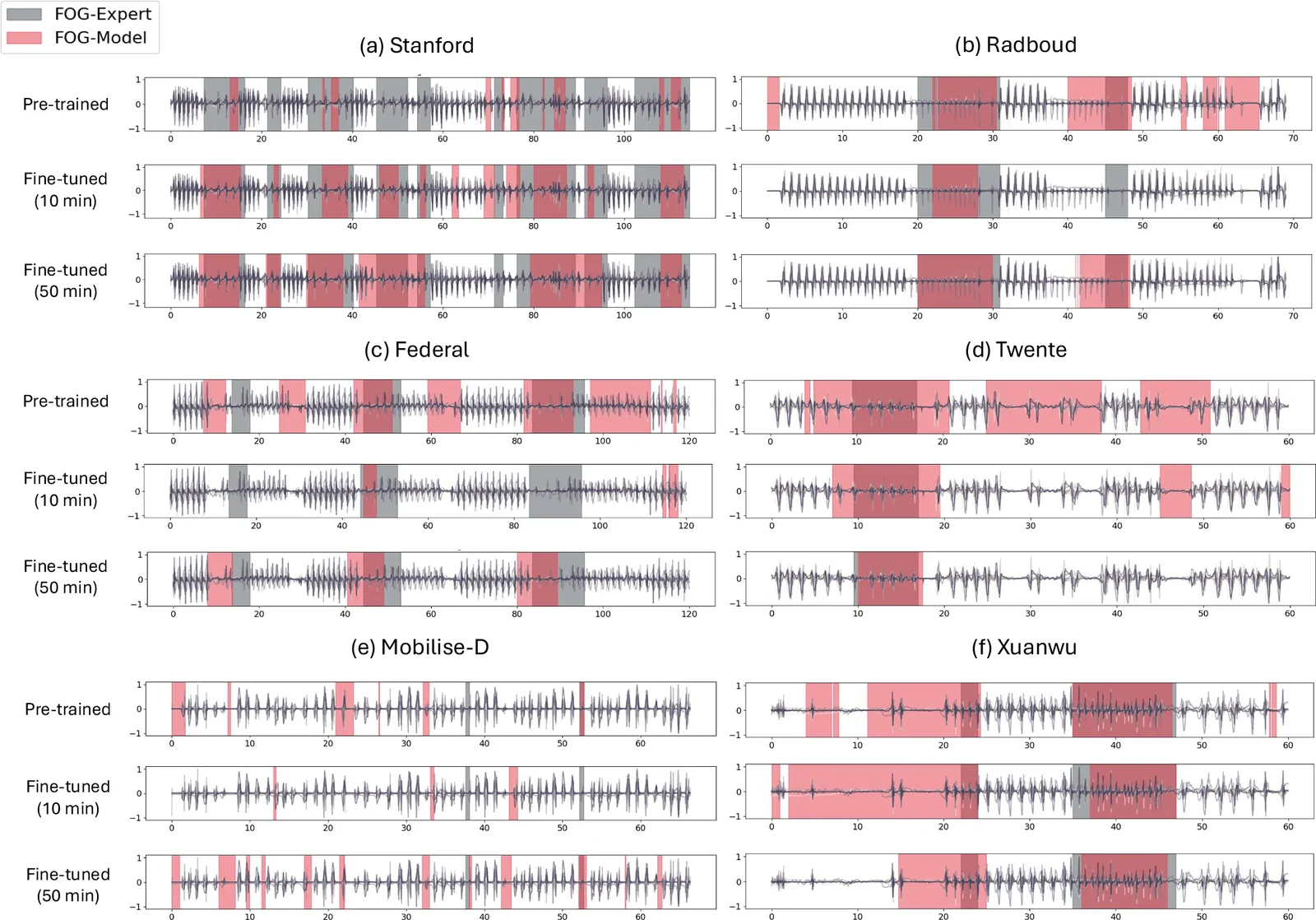
Model predictions versus expert annotations across all six external cohorts. Each panel shows one representative trial from a different external cohort, comparing three model versions against ground-truth annotations. **a** Stanford, **b** Radboud, **c** Federal, **d** Twente, **e** Mobilise-D, and **f** Xuanwu. Within each panel, three rows of annotation bars are shown from top to bottom: the pre-trained model (trained on KU Leuven data and applied without adaptation), the same model fine-tuned with 10 min of cohort-specific data, and the model fine-tuned with 50 min of cohort-specific data. In every row, gray horizontal bars represent expert annotations (ground truth) and red horizontal bars represent model-predicted FOG episodes. The normalized IMU signal is shown as a black waveform above each set of annotation rows (amplitude between −1 and 1) from a single sensor: the right foot sensor for Stanford, Radboud, and Mobilise-D; the left foot sensor for Federal; the right ankle (tibia) sensor for Twente; and the right tibia sensor for the two-IMU Xuanwu dataset. The horizontal axis represents time in seconds. FOG freezing of gait, IMU inertial measurement unit.

Figure 3a shows a Stanford example where what appears to be a shuffling-like gait pattern based on IMU characteristics was annotated as FOG by experts. These subtle IMU patterns were not initially detected by the pre-trained model but were identified after fine-tuning, though this also led to an overall increase in FPs (false positives). Figure 3b shows an example from Radboud, which includes short pre-task standing phases (e.g., before 2 s) and intermediate stopping periods (e.g., 60–65 s). The pre-trained model misclassified these as FOG, but fine-tuning enhanced its ability to distinguish volitional stops from true FOG episodes. Similarly, Fig. 3c, d, and f illustrate the model’s tendency to generate multiple FPs in datasets with only one or two IMUs, i.e., Federal, Twente, and Xuanwu, potentially due to zero-filling of missing channels to match the 5-IMU input of the pre-trained model. Fine-tuning reduced these FPs, with further improvements observed as more annotated data were used for training. Figure 3e illustrates the challenge of detecting extremely short FOG episodes in the Mobilise-D cohort, which primarily consists of mild freezers with the 360^∘^ turning-in-place task. Fine-tuning allowed the model to capture some of these brief episodes; however, the improvements were not consistent.

As discussed in previous sections, the performance drop observed when applying the pre-trained model to external datasets highlights the presence of cohort differences. While some differences, such as variations in IMU configurations (e.g., using five IMUs versus one or two IMUs), are apparent, others are more subtle. To better understand the dataset-specific differences underlying these patterns, we examined three less obvious but critical cohort differences: FOG-provoking task (Radboud and Stanford datasets), FOG annotation criteria (Stanford dataset), and variations in patient spectrum bias (Mobilise-D dataset).

**Fig. 4:**
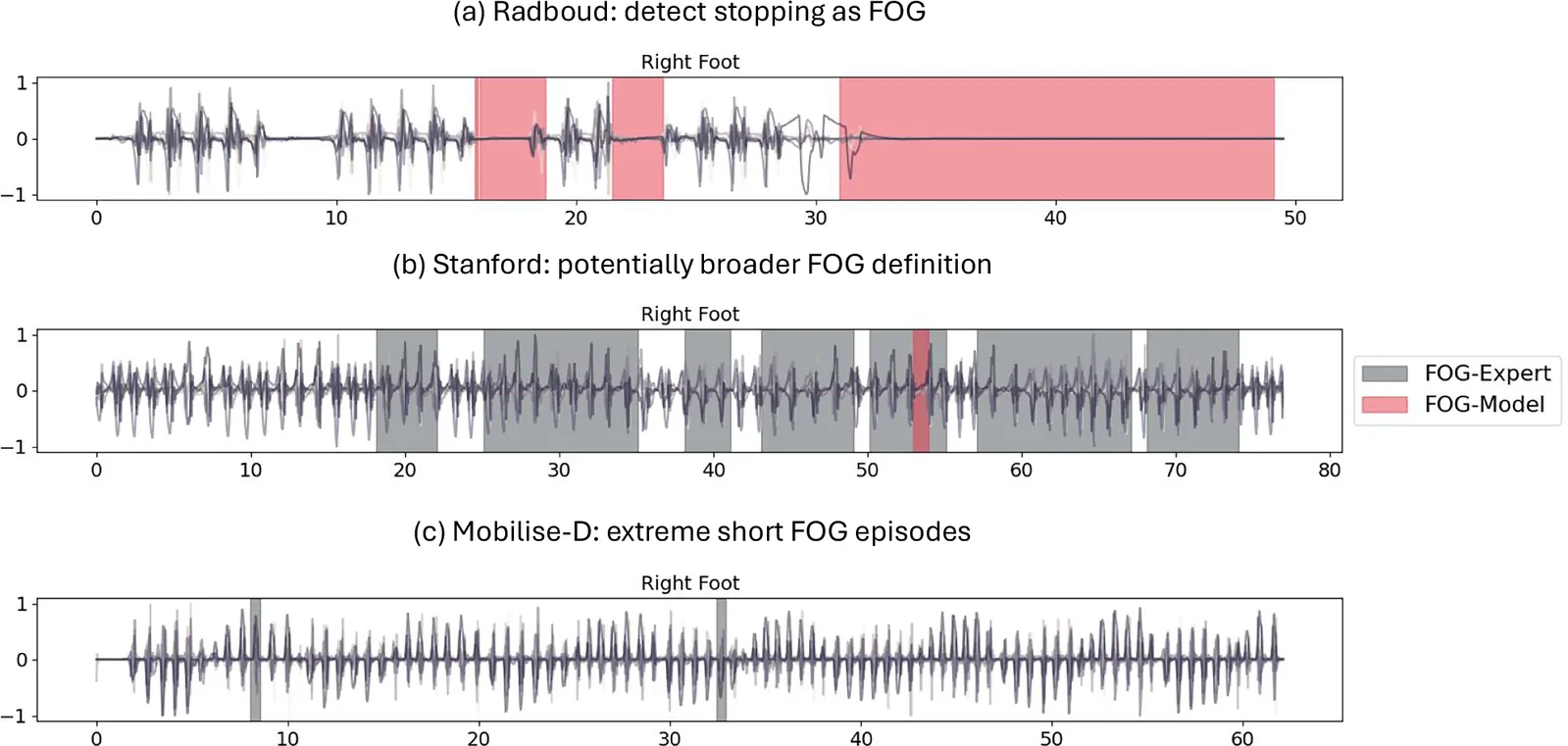
Three representative examples of cohort-specific challenges that reduce pre-trained model performance on external datasets. Each panel shows one representative trial illustrating a distinct source of cohort variability. **a** Radboud cohort: the pre-trained model misclassifies volitional stopping periods as freezing of gait (FOG) episodes, reflecting differences in the FOG-provoking task that includes intentional stops. **b** Stanford cohort: the pre-trained model fails to detect prolonged shuffling-like patterns that were annotated as FOG by the Stanford raters, reflecting differences in annotation criteria. **c** Mobilise-D cohort: the pre-trained model struggles to identify the extremely short FOG episodes typical of mild freezers during 360^∘^ turning-in-place tasks, reflecting patient spectrum bias. In all three panels, gray horizontal bars represent expert annotations (ground truth), red horizontal bars represent pre-trained model predictions, and the black waveform shows the normalized right-foot IMU signal (amplitude between -1 and 1). The horizontal axis represents time in seconds. FOG freezing of gait, IMU inertial measurement unit.

In the Radboud dataset, the pre-trained model frequently misclassified volitional stopping periods as FOG episodes, contributing to higher FP rates (Fig. 4a). To explore an intervention beyond model fine-tuning, we simulated an “expert-review” workflow in which an expert manually removed all FPs from non-FOG trials (a potential feature of the AID-FOG platform). This correction markedly improved agreement with ground truth annotations, as shown in Table 3, increasing ICC(%TF) from 0.481 to 0.893 and ICC(#FOG) from 0.098 to 0.642. These findings highlight the value of expert refinement, particularly when task-specific information (e.g., stopping periods) is documented during data collection.

In the Stanford cohort, challenges arose from differences in FOG annotation criteria. As illustrated in Fig. 4b, the Stanford dataset applied a labeling scheme that appeared to include prolonged shuffling-like episodes with observable IMU motion as FOG. These instances were not initially detected by the pre-trained model, which had been developed using a different annotation protocol. Fine-tuning allowed the model to adapt to this difference in annotation criteria, but also led to an increased #FPs (Fig. 3a). This discrepancy reflects a limitation in the original FOG definition^3^, which allowed for subjective interpretation during annotation. This challenge was also emphasized by ref. ^20^ and motivated efforts to establish a standardized definition of FOG^17,19^.

The Mobilise-D dataset presented a different challenge related to patient spectrum bias. As shown in Fig. 4c, many annotated FOG episodes were extremely brief, making them difficult to detect from the models. To assess annotation reliability, a third independent rater annotated 29 Mobilise-D trials, primarily from the Kiel and Tel Aviv center, to better reflect clinical diversity. The ICC between the fine-tuned model and the original ground truth was 0.540 for %TF and 0.756 for #FOG, whereas the ICC between the third rater and the ground truth was 0.757 and 0.543, respectively. These findings suggest that FOG detection in the Mobilise-D dataset is intrinsically challenging, even for experienced human raters. Visual comparisons of the model predictions (fine-tuned with 50 min of data), the original annotations, and the third rater’s annotations across all 29 trials are provided in Supplementary Figs. 1 and 2.

## Discussion

In this study, we developed and externally validated a DLA for assessing FOG severity using IMU data from a large cohort of people with PD (PwPD) across six independent datasets. Our results showed that while the DLA model achieved strong agreement on the local KU Leuven cohort for %TF (ICC = 0.886, CI = [0.79, 0.90]) and fair agreement for #FOG (ICC = 0.573, CI = [0.28, 0.71]), its performance dropped on the external datasets, averaging fair agreement for %TF (ICC = 0.562 ± 0.141, CI = [−0.01, 0.78]) and poor agreement for #FOG (ICC = 0.298 ± 0.270, CI = [−0.16, 0.49]). To address this generalization gap, we explored fine-tuning with expert-annotated external data across varying durations. Our analysis demonstrated that fine-tuning the model reached a plateau for both ICC(%TF) and ICC(#FOG) using 50 min of annotated data for training, with improved ICC(%TF) to 0.732 ± 0.138 (+30.2%) and ICC(#FOG) to 0.623 ± 0.214 (+108.9%). In contrast, retraining models from scratch reached a plateau with more data (90 min for ICC(%TF) of 0.708 ± 0.195 and 270 min for ICC(#FOG) of 0.528 ± 0.244). These findings highlight the data efficiency and performance benefits of fine-tuning, which enables better generalization with less annotated data. It is important to note that these durations reflect the amount of trial data rather than clinician annotation time.

However, fine-tuning alone cannot replace expert review at the current stage, since our best ICCs still lie at the lower boundary of reported inter-rater ranges (0.730-0.990 for %TF and 0.390-0.950 for #FOG^10,15,34,41–43^). Though it should be noted that this comparison is not fully equivalent: inter-rater studies are conducted under controlled conditions with harmonized training and shared protocols within a single study context, whereas DLAs are deployed across external datasets with differing protocols, sensor setups, and clinical conventions. The inter-rater range thus represents an aspirational benchmark rather than a directly comparable reference. Regardless, we consider that fully automated FOG detection is not yet feasible due to the lack of unified standards and the resulting variability across studies, such as sensor configurations, task design, and annotation criteria. While unified standards are being developed^17,19,20,23^ and while patient spectrum heterogeneity cannot be fully resolved through standardization alone, progressive model improvement with fine-tuning can already provide a temporary solution to make the annotation workload more efficient, since only a subset of data (e.g., 50 min) requires manual annotation. In our proposed AI-assisted workflow (Fig. 1c and the Supplementary Information), experts would first review model-generated annotations to (1) flag any missed FOG episodes and (2) remove clear FPs. Those corrections could be further used to fine-tune the model, shifting its decision boundary to catch previously missed FOG episodes and dropping FPs. A practical use case would imply uploading pilot data to generate customized models for clinical trials. Nevertheless, the AID-FOG platform currently represents a proof-of-concept, and its clinical utility remains to be formally evaluated. For example, we fine-tuned solely on existing ground-truth labels and did not incorporate expert-adjusted annotations; in practice, initial exposure to model predictions may bias experts toward correcting only the most obvious errors. Furthermore, while 50 min of trial data were sufficient to reach a performance plateau, the actual clinician time required to annotate this data, and whether the resulting model performance satisfies end-users in practice, remain open questions. Therefore, future user studies with independent raters should evaluate whether fine-tuning on expert-refined annotations reproduces or improves the current performance. Equally, the annotation bias introduced by selective correction and the clinician time required to annotate sufficient fine-tuning data need to be quantified, and it needs to be determined at what performance level experts find the model output satisfactory.

In this study, we assessed both fine-tuning and training-from-scratch using 7-fold participant-level cross-validation: holding out one fold for testing each run and averaging results to avoid split-dependent effects. Within each training fold, we built nested annotation sets of increasing duration (10, 20, ..., 360 min) by sequentially adding participants while preserving the cohort’s overall %TF distribution. This design ensures that performance gains reflect added annotation time rather than changes in participant mix or FOG prevalence, and mirrors the human-in-the-loop workflow of incrementally uploading extra minutes from the same patients. However, note that cohorts differed in their recording lengths per participant. For example, with a 50-min fine-tuning, Stanford, which contains more trials per participant, could include five 10-min segments per participant with a total of five participants; whereas Mobilise-D can be assembled from 50 one-minute trials across 50 participants. Although this does not specify how many participants must be recruited or re-annotated, our approach quantifies the exact number of minutes of data needed to reach any target performance level. Although fine-tuning plateaus at just 50 min of annotated data, which still requires non-trivial annotation effort, this is considerably less burdensome than training from scratch, which required 90 min to plateau for %TF and up to 270 min for #FOG.

Additionally, detecting FOG in the predominantly mild-freezer Mobilise-D cohort was particularly challenging, illustrating the risks of applying models trained on severe freezers to mildly affected populations. Although Mobilise-D episodes are shorter on average (mean 0.98 s vs. 3.19 s in KU Leuven), the KU Leuven training data also contains short episodes with a similarly right-skewed duration distribution, suggesting that differences in patient spectrum, rather than episode duration alone, drive the limited generalization to this cohort. We provide a visualization of the FOG episode duration distribution in Supplementary Fig. 3. Although fine-tuning can increase performance in this group, the improvements are smaller, and the ceiling remains lower than in severe-freezer cohorts. Until sufficiently large datasets of mild and pre-freezers exist, expert reviews are essential, and an AI-assisted workflow could potentially benefit this process. Looking further ahead, motion foundation models pre-trained on large and diverse gait datasets, such as CARE-PD^44^, may offer greater robustness to such distribution shifts.

Moreover, as the primary goal of this study was to evaluate model generalization across the heterogeneous conditions already present in the FOG detection field, existing datasets with their inherent variability were included. Beyond disease and FOG severity compared across cohorts via NFOG-Q and MDS-UPDRS III scores, patient-level factors such as medication profiles^45^, comorbidities^46^, cultural differences and assessment time of day^22^ were not systematically recorded, given the retrospective design, and may contribute to residual variability in model performance. Notably, these factors are rarely standardized across existing FOG detection studies, and their influence on automated FOG detection remains largely unexplored. Future studies should systematically capture these variables to better understand what drives generalization across diverse populations.

Compared to other large-scale efforts, Salomon et al. ran a machine learning challenge using over 90 h of daily-living accelerometer data from a single IMU, training on 833 trials from 62 participants and 91 trials from 38 participants with PD, with public (945 trials, 26 participants) and non-public test sets (391 trials, 14 participants)^22^. The top five DLAs all incorporated self-attention or transformer blocks, consistent with our finding that LTContext, with attention modules, outperforms MS-TCN and ASFormer in FOG detection on external datasets. The top-performing model in their study achieved a recall of 0.78 and a specificity of 0.94, with a strong agreement for %TF (ICC > 0.87) and fair agreement for #FOG (ICC > 0.50). These results exceed those we observed when evaluating our models on the external cohorts. However, it is important to note that although their data came from three centers (KU Leuven, Harvard, and Tel Aviv), their train-test split minimized cohort heterogeneity, such as differences in task protocols, sensor configurations, and annotation criteria between training and evaluation sets. Borzí et al. evaluated a single-ankle IMU model on 22 participants (101 FOG episodes) and externally validated it on two independent datasets (545 episodes), achieving 88–95% detection sensitivity but suffering from high FP rates^47^. Similarly, in our internal-to-external validation, we observed an increase in #FPs on external datasets. However, after fine-tuning, the #FPs decreased, and both specificity and precision improved (see the Supplementary Information).

With the updated FOG definition now available^19^, re-annotating existing datasets to align with new standards would require substantial effort. However, as this update represents a shift in annotation criteria, similar to the differences we observed in the Stanford cohort, our fine-tuning approach can adapt pre-trained models to future definitions without retraining models from scratch. Similarly, fine-tuning could possibly streamline this process by progressively improving model adaptation to future definitions. Ultimately, the ICFOG is aiming to standardize FOG annotation criteria^17,19^, IMU sensor protocols, and FOG-provoking tasks^20^, which could help reduce inter-center variability. However, whether this translates to improved model generalization remains to be demonstrated by future studies.

In conclusion, we presented a DLA trained on a large local cohort within KU Leuven and externally validated the model on six external cohorts of people with PD and FOG. Although performance dropped due to cohort differences, fine-tuning with limited external data (e.g., 50 min) substantially improved generalizability, albeit not yet to optimal levels, underscoring the continued need for expert review. To support both trust and model adaptation, we proposed an AI-assisted, human-in-the-loop workflow: the DLA suggests FOG episodes, experts correct and refine these annotations, and the model is iteratively fine-tuned. Finally, we are building the AID-FOG platform, an interactive platform tailored for clinical annotation and model updating. Together, these contributions represent important steps toward making AI-assisted FOG analysis in PD more robust, clinically meaningful, and scalable across diverse real-world settings.

## Methods

### Study design and participants

This multicentre retrospective study developed a DLA, trained on a local cohort (KU Leuven) and externally validated on six cohorts. Data were collected from eight universities: KU Leuven (non-public), Radboud University (non-public)^33^, Stanford University (public)^34^, Federal University of ABC (public)^35^, University of Twente (public)^36,37^, Beijing Xuanwu Hospital^38,39^, and the Mobilise-D project (non-public)^13^, which included data from KU Leuven, University Medical Center Schleswig-Holstein Campus Kiel, and Tel Aviv Sourasky Medical Center. We included only public datasets that provide lower-limb IMUs with both accelerometer and gyroscope data collected during controlled tasks; datasets offering only accelerometers^22,48^ or only upper-limb sensors^49^ were excluded.

- **KU Leuven Cohort (Local Dataset)**: Included a total of 85 PwPD with FOG across four datasets. IMU configurations and FOG-provoking tasks varied across datasets:
  - Dataset 1: Included 40 PwPD (30 self-reported freezers, 10 non-freezers)^27^, with 2 participants excluded due to technical failure. Tasks: Timed Up and Go (TUG) test and one-minute alternating 360^∘^ turns under both OFF ( > 12 h since last dose) and ON (≥1 h post-intake) medication states, each performed with and without a cognitive dual task (i.e., an auditory Stroop task^50^) and with and without a volitional stopping period between tasks. Used five Shimmer3 IMUs (64 Hz, Shimmer Research, Ireland) placed on the pelvis and both sides of the tibia and talus.
  - Dataset 2: Included 28 PwPD (14 freezers, 14 non-freezers)^31^, with 1 excluded due to technical issue. Tasks: 5m straight-line walk with/without 180^∘^/360^∘^ turns under OFF-medication. Used synthesized IMUs virtually placed at the same positions as dataset 1, derived from Vicon motion capture (100 Hz, 8-camera system, Vicon, UK)^51,52^.
  - Dataset 3: Included 8 PwPD with baseline and 12-month follow-up data (out of 17)^32^; all trials from both visits for each participant were combined (not entered as separate participants). Tasks: straight walking and 360^∘^ turns under OFF-medication. Used synthesized IMUs virtually placed at the same positions as dataset 1, derived from Vicon motion capture (100 Hz, 8-camera system, Vicon, UK)^51,52^.
  - Dataset 4: Included 12 PwPD with a walking/slalom task (three walking/slalom bouts separated by stops) under ON-medication, each completed with and without a cognitive dual task. Used five OPAL IMUs (128 Hz, Clario, USA) placed as dataset 1. Note that this dataset originates from an ongoing study (ClinicalTrials.gov NCT05511597).
- **Radboud University Cohort (External Dataset 1)**^33^: Included 25 PwPD with FOG. Used 17 Xsens Awinda IMUs (60 Hz, Xsens Technologies, Netherlands). IMUs covered full-body motion capture. Tasks: 15m walking with volitional stopping, walking straight, and auditory-cued 360^∘^ turns. Conducted under OFF-medication.
- **Stanford University Cohort (External Dataset 2)**^34^: Included 7 PwPD with FOG, each completing 5-14 repetitions of an OFF-medication slalom course (two ellipses and two figure-eight turns around barriers) while wearing seven OPAL IMUs (128 Hz, Clario, USA) at the chest, lumbar spine (pelvis), ankles (tibia), and feet (talus); data from multiple visits (up to 44 months apart) were aggregated per participant rather than treated as separate participants.
- **Federal University of ABC Cohort (External Dataset 3)**^35^: Included 35 PwPD with FOG. Used 1 Physilog 5 IMU (128 Hz, Gait Up, Switzerland) placed on the left lower leg (tibia position). Task: two-minute alternating 360^∘^ turn. Conducted under ON-medication.
- **University of Twente Cohort (External Dataset 4)**^36,37^: Included 21 PwPD with FOG. Used 1 Movisens Move 4 IMU (60 Hz, movisens GmbH, Germany) mounted on the right ankle (tibia position). Data were recorded in semi-free-living conditions, encompassing daily routines (e.g., cleaning, setting the table, mopping, making the bed), outdoor walking, and a clinical assessment. The full dataset was divided into “all movements" and “walking-and-turning" segments; this study analyzed only the walking-and-turning portion. All recordings were performed under OFF-medication.
- **Beijing Xuanwu Hospital (External Dataset 5)**^38,39^: Included 12 PwPD with FOG. A single TDK MPU6050 IMU (100 Hz, TDK InvenSense, USA) was mounted on the left or right tibia (or both). Two walking-and-turning tasks were performed: Participants stood from a chair, walked 5 m into an 18 m corridor, navigated three obstacles by rotating around them, made a 180^∘^ turn at the far end, then retraced their steps back to the chair and sat down; Participants walked 3 m from the chair to a 0.6 m square, executed a 180^∘^ turn inside the square, and returned to sit. All trials were conducted under OFF-medication. Both raw and filtered data are available in this public dataset; since the raw data include signals recorded during patient preparation, we used only the filtered data in this study. Detailed processing steps are described in the original publications.
- **Mobilise-D Project Cohort (External Dataset 6)**^13^: Included 52 PwPD from KU Leuven, 90 PwPD from University Medical Center Schleswig-Holstein Campus Kiel, and 35 PwPD from Tel Aviv Sourasky Medical Center (after excluding 6, 9, and 6 participants respectively for technical issues). Used OPAL IMUs (128 Hz, Clario, USA) at the pelvis and both sides of the tibia and talus. Task: one-minute alternating 360^∘^ turns with a concurrent cognitive dual task. Conducted under ON-medication. Note: to represent mild-to-moderate freezers, the KU Leuven subset was merged with the Kiel and Tel Aviv cohorts into a single external cohort (termed Mobilise-D).

All cohorts included synchronized video recordings for FOG annotation. The KU Leuven datasets were annotated by two external raters using the criteria: “FOG was defined as a brief episode with the inability to produce effective steps”^3^. Specifically, an episode started only when the participant’s foot suddenly ceased producing an effective forward step and displayed FOG-related features, and ended only after at least two effective steps had been completed (these two steps were not included in the episode)^53^. The Stanford dataset was annotated by two experienced raters using the criteria: “a FOG event was defined by loss of alternating stepping, complete cessation of forward motion, or trembling of the legs”^54^. The Federal dataset was annotated by two experienced raters using the criteria: “The beginning of a FOG episode was defined when the turn pattern (alternating right and left steps) was arrested, or when participants appeared to be trying unsuccessfully to initiate or continue the turn. The end of the episode was marked by the execution of an effective step followed by continuous turning”^41^. The Mobilise-D dataset was annotated by two independent expert raters and verified by a third experienced moderator in cases of disagreement. Each FOG episode was further labeled as either predominantly “trembling”, characterized by oscillatory movements between 3-8 Hz visible in the legs for more than 50% of the episode, or predominantly “akinesia”, defined by an absence of visible leg trembling and a lack of foot progression for more than 50% of the episode. The Radboud and Twente cohorts were annotated by two experienced raters following the clinical definition provided by ref. ^3^: “FOG was defined as a brief, episodic absence or marked reduction of forward progression of the feet despite the intention to walk.” The Xuanwu cohort was annotated by two physicians^38^, although the specific criteria used to label FOG episodes were not reported.

Clinical assessments were available in all cohorts except Stanford, including:

- Movement Disorders Society Unified Parkinson’s Disease Rating Scale (MDS-UPDRS III)^55^
- New Freezing of Gait Questionnaire (NFOG-Q)^56^ (Xuanwu cohort included the Freezing of Gait Questionnaire (FOGQ) instead^57^)

Some cohorts also included:

- Hoehn & Yahr (H&Y) Scale^58^
- Mini-Mental State Examination (MMSE)^59^
- Montreal Cognitive Assessment (MoCA)^60^

All original data collections were approved by their respective local ethics committees at the time of data collection and performed in accordance with the Declaration of Helsinki, with written informed consent obtained from all participants. Original approvals were obtained as follows: the KU Leuven cohort by the Research Ethics Committee UZ/KU Leuven S65059, S65984 and S64977 for the Mobilise-D Project Cohort, along with approvals from Kiel University (D 630/20), and the Edmond J. Safra Center for Ethics at Tel Aviv University (0551-19-TLV); the Radboud cohort by the Medical Ethics Committee Twente^33^; the Twente cohort by the Ethics Review Board (CMO) Arnhem-Nijmegen (N171352.09L.L9)^37^; the Federal University of ABC cohort by the Federal University of ABC^35^; the Beijing Xuanwu Hospital cohort by the Ethics Committee of Xuanwu Hospital, Capital Medical University (2019-014)^38^; and the Stanford cohort by the Stanford University Institutional Review Board^34^.

### Data harmonization

To harmonize the datasets, we first applied a zero-phase fourth-order Butterworth low-pass filter with a 16 Hz cutoff frequency, selected to preserve the locomotor (0–3 Hz) and freezing (3–8 Hz) bands^61^. All IMU data were then resampled to a uniform 32 Hz sampling frequency. Further preprocessing was performed using the gaitmap Python library^62^, which provides automated IMU signal processing tools. While its algorithms are primarily designed for foot-worn IMUs, they were used to ensure consistent sensor alignment across datasets with similar placements.

First, gaitmap’s gravity alignment algorithm was applied, rotating signals so that the gravitational component is fully captured by the *z*-axis. A value of 9.81 was then subtracted to center all signals around zero. This step was skipped for datasets where the gravitational component was already removed. Next, IMU signals were aligned using gaitmap’s PCA-based algorithm, which detects the medio-lateral component as the first principal component and rotates the signals accordingly. Since PCA sign orientation depends on data distribution, an additional correction was applied to prevent a 180^∘^ misalignment of accelerations. This final correction was performed using gaitmap’s trajectory reconstruction algorithm. Lastly, the standard offset was removed by subtracting the mean of each signal before feeding the data into the model.

As most datasets included five IMUs, adaptations were required for the Federal, Twente, and Xuanwu datasets, which each employed either one or two IMUs. To maintain consistent input dimensions across all datasets, missing IMU channels for these three datasets were filled with zeros during preprocessing.

The Twente dataset underwent preprocessing as part of its original public release, where signals were segmented into 120 time-step windows with an 87.5% overlap for FOG segments and a 50% overlap for non-FOG segments. To ensure consistency with the other datasets, the segmented windows were reorganized into a continuous sequence for analysis. Unlike other datasets, where tasks lasted less than 2 min, the Twente dataset included longer semi-free-living walking and turning sequences. For consistency in processing across cohorts and to prevent memory overload, these extended trials were partitioned into 2-min segments before model training.

### Problem statement

Formally, the IMU data of each gait task can be represented as a 2D matrix $X\in {{\mathbb{R}}}^{T\times N{C}_{in}}$, where *T* represents the number of samples (duration of the gait task), *N* the number of IMUs, and *C*_*in*_ the number of features per IMU (3-axis accelerometer and 3-axis gyroscope). Similarly, the ground truth can be represented as a 2D matrix $Y\in {{\mathbb{R}}}^{T\times L}$, where *T* represents the number of samples and *L* the number of classes (i.e., *L* = 2 for FOG and non-FOG).

### Deep neural network design

Fine-grained FOG detection often suffers from over-segmentation, i.e., splitting true FOG episodes into multiple short segments, which complicates expert review in AI-assisted annotation tools such as the AID-FOG platform. MS-TCN mitigates this issue by stacking TCN stages with a smoothing loss to enforce temporal consistency^28,63^. Building on MS-TCN, ASFormer^29^ and LTContext^30^ integrated attention modules (recently shown to increase FOG detection performance^22^) to capture long-range dependencies and further refine action segmentation. Rather than proposing a new architecture, we systematically evaluated MS-TCN, ASFormer, and LTContext to identify the best-performing approach for FOG detection across heterogeneous external cohorts.

**Fig. 5:**
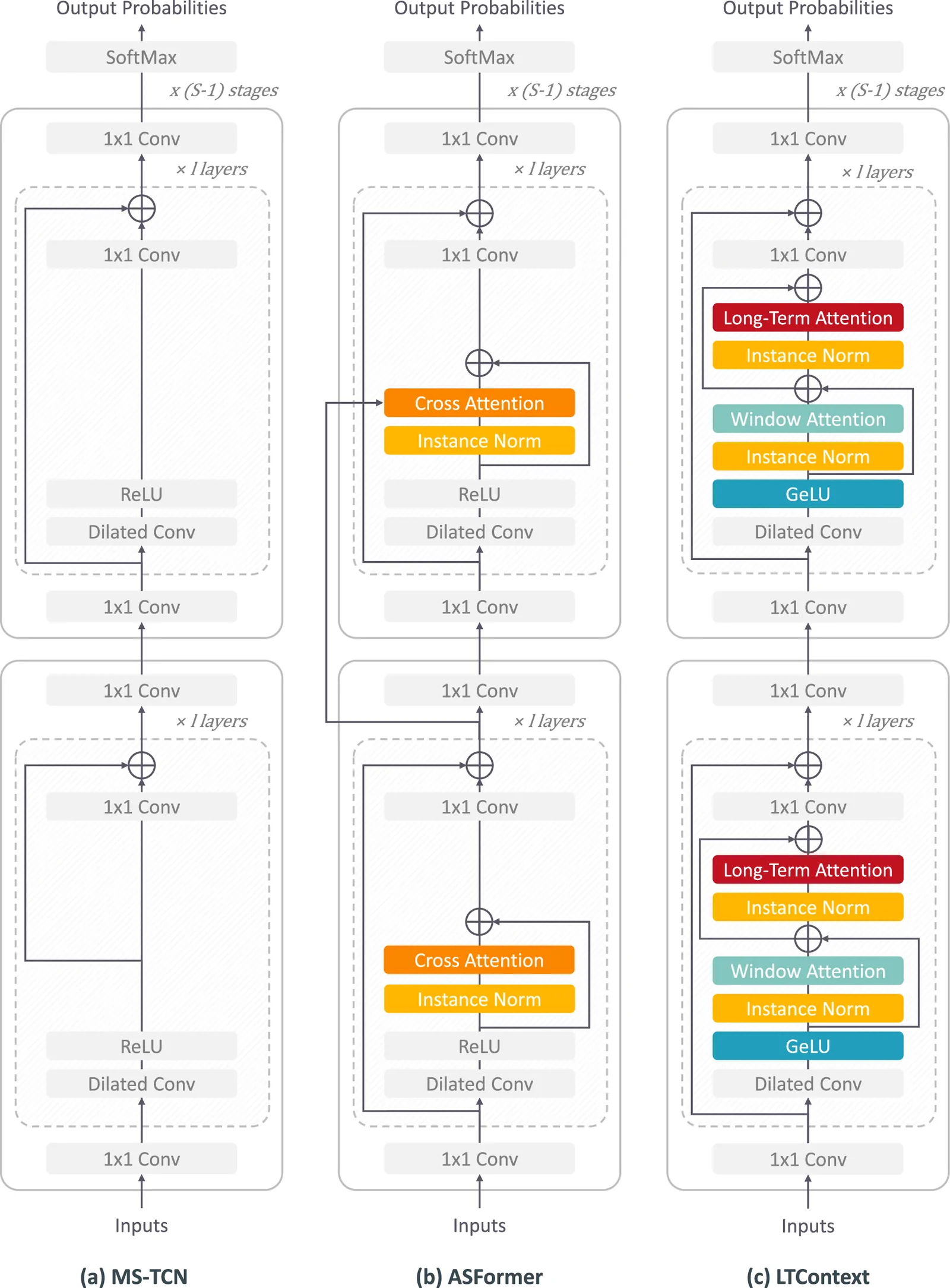
Architectures of the three sequence learning models evaluated for FOG detection. The figure compares three state-of-the-art action-segmentation networks, all sharing a common backbone of an input projection layer, an initial prediction stage, and *S* − 1 refinement stages. **a** Multi-Stage Temporal Convolutional Network (MS-TCN): each stage contains l dilated temporal convolution layers with rectified linear unit (ReLU) activations and residual skip connections. **b** Action Segmentation Transformer (ASFormer): extends MS-TCN by adding instance normalization (yellow blocks) and a self-attention layer in the first (encoder) stage or a cross-attention layer (orange blocks) in subsequent (decoder) stages. **c** Long-Term Context Action Segmentation model (LTContext): replaces ReLU with Gaussian error linear unit activations (GeLU; cyan blocks), and augments each temporal convolution layer with a windowed local attention (green blocks) and a sparse long-term attention (red blocks), each preceded by instance normalization (yellow blocks). In all three architectures, dashed outlined boxes represent layers that are repeated *l* times within each stage, and the full stage block is repeated *S* − 1 times as refinement stages. The final SoftMax layer produces per-sample class probabilities. Conv convolution, ReLU rectified linear unit, GeLU Gaussian error linear unit, MS-TCN Multi-Stage Temporal Convolutional Network, ASFormer Action Segmentation Transformer, LTContext Long-Term Context action segmentation model.

All three models share a common structure (see Fig. 5): an input projection layer (1 × 1 convolution, i.e., a learned linear mapping applied independently at each time step) maps ${\bf{X}}\in {{\mathbb{R}}}^{T\times N{C}_{in}}$ to a hidden representation ${f}_{adj}\in {{\mathbb{R}}}^{T\times C}$, followed by an initial prediction stage and *S* − 1 refinement stages, each producing class probabilities:

1 $${\widehat{{\bf{Y}}}}^{(s)}=\zeta \left({f}_{h}^{(s)}\right)\in {{\mathbb{R}}}^{T\times L},\,s=0,\ldots ,S-1,$$

where ${f}_{h}^{(s)}\in {{\mathbb{R}}}^{T\times C}$ is the block output at stage *s* (derived from *f*_*adj*_ for *s* = 0, and from ${\widehat{{\bf{Y}}}}^{(s-1)}$ for *s* > 0) and *ζ* denotes the SoftMax function. The stage losses are summed to compute the final loss (section “Loss function”).

The models differ in their per-stage block design:

- **MS-TCN**^28^ processes each stage through *l* dilated TCN layers with residual skip connections and ReLU activations.
- **ASFormer**^29^ augments each TCN layer with Instance Normalization (to stabilize training) and a self-attention layer in the first stage (encoder) or a cross-attention layer attending to the encoder output in subsequent stages (decoder), enabling the model to capture global temporal context.
- **LTContext**^30^ replaces ReLU with GeLU and further augments each TCN layer with windowed attention for local context and sparse long-term attention for global context, each preceded by Instance Normalization. In stage 1, both attention mechanisms attend to the input features; in further stages, they attend to predictions ${\widehat{{\bf{Y}}}}^{(s-1)}$ from the previous stage.

To develop the best possible model, first, for each model, optimal hyperparameter values (hidden representation dimension *C*, number of layers *l*, number of stages *S*) are found using the internal KU Leuven dataset with 7-fold cross-validation. In the next step, the three models are trained on the full KU Leuven dataset with the best hyperparameters. Then we perform inference on all the external datasets, and ICC(%TF) and ICC(#FOG) are computed per dataset. Lastly, the optimal model is selected with the best mean ICC(%TF) and ICC(#FOG) on the external datasets.

### Loss function

A loss function (${\mathcal{L}}$) computed the sum of the losses over all *S* stages:

2 $${\mathcal{L}}=\mathop{\sum }\limits_{s=0}^{S-1}{{\mathcal{L}}}^{s}$$

where ${{\mathcal{L}}}^{s}$ represents the loss for the output probabilities at stage *s*. Each stage’s loss combines the cross-entropy loss (${{\mathcal{L}}}_{cls}$) and the smoothing loss (${{\mathcal{L}}}_{T-MSE}$), with the latter weighted by a hyperparameter *λ*_*T*−*MSE*_ that controls its contribution. In this study, *λ*_*T*−*MSE*_ is set to 0.15, as recommended in previous research^28^.

The loss for each stage (*L*^*s*^) is formalized as:

3 $${{\mathcal{L}}}^{s}={{\mathcal{L}}}_{\,{\rm{cls}}}^{s}+{\lambda }_{{\rm{T}}-{\rm{MSE}}}\,{{\mathcal{L}}}_{\,{\rm{T-MSE}}}^{s}$$

The cross-entropy loss measures classification performance and is defined as:

4 $${{\mathcal{L}}}_{{\rm{cls}}}=-\frac{1}{T}\mathop{\sum }\limits_{t,l}{y}_{t,l}\log ({\widehat{y}}_{t,l}),$$

where *T* is the sequence length, *L* is the total number of classes, *y*_*t*,*l*_ is the ground truth for class *l* at time step *t*, and ${\widehat{y}}_{t,l}$ is the predicted probability of class *l* at time *t*. The smoothing loss, ${{\mathcal{L}}}_{{\rm{T}}-{\rm{MSE}}}$, is a truncated mean squared error applied to the log-probabilities of consecutive samples:

5 $${{\mathcal{L}}}_{{\rm{T}}-{\rm{MSE}}}=\frac{1}{TL}\mathop{\sum }\limits_{t,l}{\widetilde{\Delta }}_{t,l}^{2},$$

where $${\widetilde{\Delta }}_{t,l}$$ is:

6 $${\widetilde{\Delta }}_{t,l}=\left\{\begin{array}{ll}{\Delta }_{t,l}, & \,{\rm{if}}\,{\Delta }_{t,l}\le \tau ,\\ \tau , & \,{\rm{otherwise}}\,.\end{array}\right.$$

Here, *Δ*_*t*,*l*_ represents the absolute difference in log-probabilities between consecutive time steps:

7 $${\Delta }_{t,l}=| \log ({\widehat{y}}_{t,l})-\log ({\widehat{y}}_{t-1,l})| ,$$

and *τ* is a hyperparameter defining the threshold for truncating the smoothing loss.

### Evaluation and training strategies

Hyperparameters for the LTContext model were selected using 7-fold participant-level cross-validation on the KU Leuven cohort. We tuned batch size, number of layers, number of stages, and number of hidden features sequentially, at each step selecting the configuration that produced the highest F1 and/or F1@50 on FOG trials when statistically significant differences (*p* < 0.05) were observed, and otherwise selecting the configuration with the highest ICC for %TF. The same procedure was applied to MS-TCN and ASFormer for the architecture comparison. Full hyperparameter search results and the final selected configurations are reported in the Supplementary Information (Supplementary Tables 1–5). The final LTContext configuration included a batch size of 1, 10 layers, 4 stages, and 64 hidden features, while other hyperparameters remained as specified in the original LTContext publication^30^.

The model was first pre-trained on the KU Leuven cohort using the Adam optimizer (*β*_1_ = 0.9, *β*_2_ = 0.999)^64^ for 50 epochs. Training began with a learning rate of 0.00025, which was gradually reduced to 0.00005 using a cosine decay schedule^30^.

We evaluated two strategies for adapting the model to external cohorts: (1) training from scratch and (2) fine-tuning the pre-trained model. Both strategies used 7-fold participant-level cross-validation. In each fold, we sampled training durations in 10-min increments up to 60 min (to match Stanford’s maximum) and in larger steps thereafter (90, 120, 150, ..., 360 min), as a compromise between temporal resolution and computational feasibility. This setup required 7 folds × 5 cohorts × 2 strategies × 16 time points = 1120 separate model trainings. The maximum available durations varied by cohort (Stanford: 60 min; Mobilise-D: 150 min; Federal: 120 min; Radboud and Twente: 360 min). For reporting averaged results, we used each cohort’s last available time point, assuming performance generally improves or plateaus with more data^65^. We defined the plateau as the smallest training duration beyond which additional data yields no more than a one-percentage-point gain in the target metric (e.g., ICC(%TF))^66^.

For models trained from scratch, all hyperparameters and optimization settings mirrored those of pre-training, including training for 50 epochs using Adam and cosine learning rate decay.

Fine-tuning followed a selective learning rate strategy inspired by ref. ^67^. The input projection and LTContext blocks (feature extraction layers) were fine-tuned with a learning rate of 0.00025, while the prediction layers (output projections and refinement stages) were updated with a higher learning rate of 0.0025 to accelerate adaptation. Optimization used the Adam optimizer (batch size = 1) with cosine decay to 0.00005. For fine-tuning, we used a fixed number of backpropagation steps rather than a fixed number of epochs, because fixed-step schedules provide more precise control over the extent of model updates and ensure stable convergence when fine-tuning across datasets of varying sizes. The number of steps was fixed at 3000 for all fine-tuning runs, based on a convergence analysis on the 360-min condition (mean 2920.71 ± 1050.34 steps to early-stopping convergence with patience of five epochs across the 7-fold cross-validation; full details in the Supplementary Information). All other hyperparameters matched the pre-training configuration.

### Metrics and statistics

The primary metric for evaluating action segmentation reliability was the sample-wise F1 score. To assess temporal consistency, we additionally used the segment-wise F1 score with a 50% intersection-over-union threshold (F1@50)^68^, which penalizes over-segmentation by requiring predicted segments to closely align with ground truth annotations^69^. The segment-wise F1@50 is particularly relevant for the proposed AID-FOG platform, as lower F1@50 scores imply that predicted FOG episodes are temporally misaligned, requiring more manual adjustments by clinicians to refine segment boundaries. To ensure a detailed and unbiased evaluation of model performance, we reported metrics separately for FOG and non-FOG trials. For trials containing expert-annotated FOG episodes (FOG trials), both the sample-wise F1 and segment-wise F1@50 were computed. For non-FOG trials, performance was assessed using the following metrics:

- Mean number of false positive (FP) episodes per trial (#FP per trial)
- Mean FP duration per trial

**Fig. 6:**
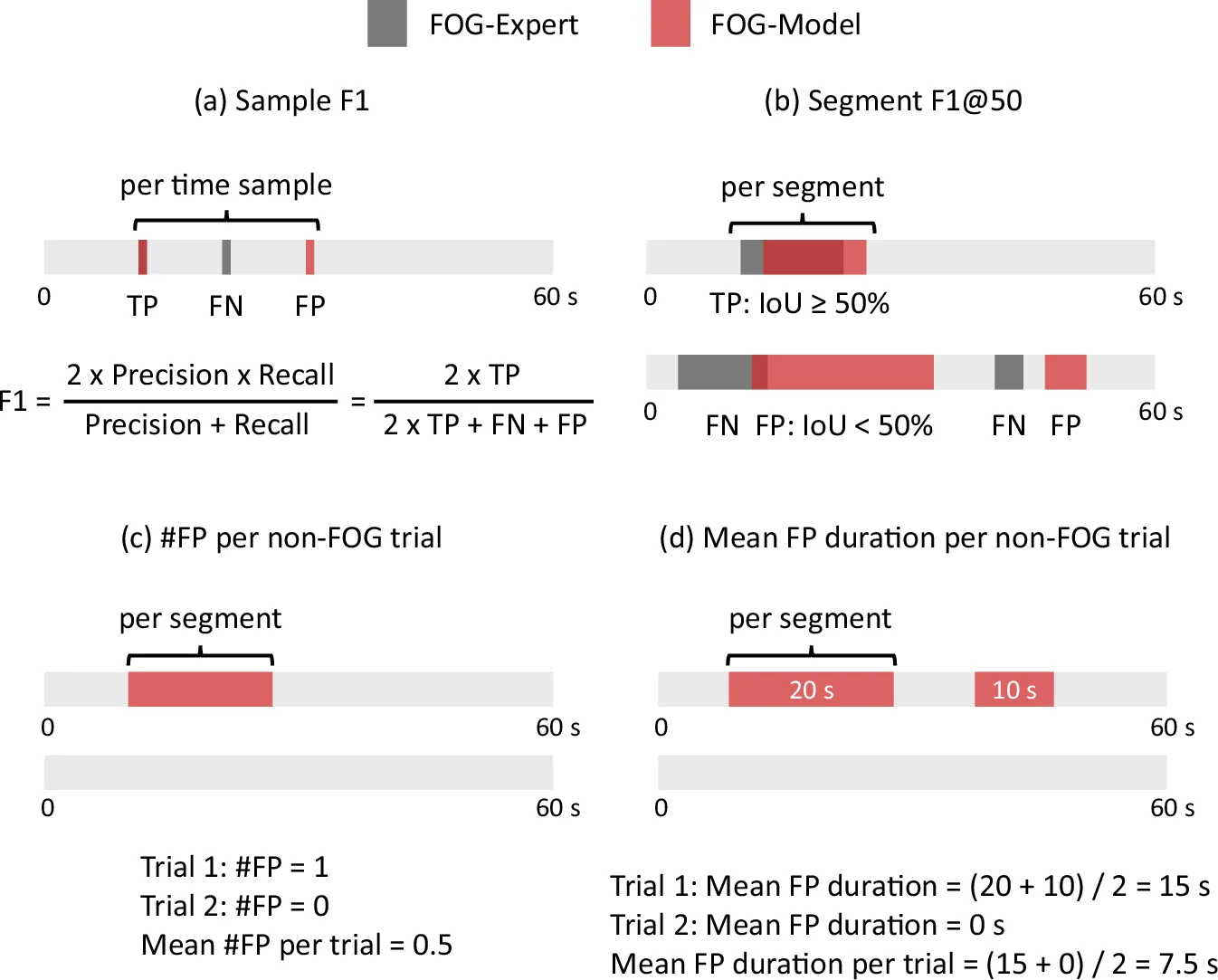
Illustration of the four evaluation metrics used to assess freezing of gait detection performance. This figure depicts how each of the four metrics is computed on simulated expert (gray bars) and model (red bars) annotations. **a** Sample-wise F1 score, computed per time sample on freezing of gait (FOG) trials by classifying each sample as true positive (TP), false negative (FN), or false positive (FP) and combining precision and recall into a single harmonic mean. **b** Segment-wise F1@50, computed per segment on FOG trials, where a predicted segment counts as a true positive only if its intersection-over-union (IoU) with a ground-truth segment is at least 50%; otherwise, it contributes a false positive (FP) and the unmatched ground-truth segment contributes a false negative (FN). **c** Mean number of false-positive episodes per non-FOG trial (#FP per trial), computed by counting predicted FOG segments within trials that contain no ground-truth FOG and averaging across trials. **d** Mean false-positive duration per non-FOG trial, computed by summing the durations of all predicted FOG segments within each non-FOG trial and averaging across trials. In all panels, gray bars represent expert annotations and red bars represent model predictions; the horizontal axis represents time in seconds. FOG freezing of gait, TP true positive, FN false negative, FP false positive, IoU intersection-over-union, #FP number of false-positive episodes.

All metrics were first computed at the trial level, then averaged across trials within each participant to obtain a participant-level score, and finally averaged across participants to obtain the reported mean ± SD. Lower values for both #FP per trial and mean FP duration indicate higher precision, reflecting fewer and shorter erroneous FOG detections. These metrics are also practically meaningful for the AID-FOG platform, as more FPs translate to more manual clicks and corrections by annotators. Figure 6 illustrates the computation of these four evaluation metrics. To align with prior FOG detection research^20^, we also computed the traditional machine learning metrics, e.g., accuracy, recall, specificity, and precision, across the whole dataset of each external cohort. The results of these metrics are detailed in Supplementary Table 7.

FOG severity was quantified using the number of FOG episodes (#FOG) and the percentage of time spent frozen (%TF), defined as the cumulative duration of all FOG episodes divided by the total duration of the gait task^15^. To assess reliability in FOG severity estimation, we computed the intraclass correlation coefficient (ICC) using a two-way random effects model (ICC(2,1)), reflecting absolute agreement for a single measurement.

All metrics were computed at the participant level by aggregating all gait tasks per participant. The statistical significance of F1-score differences between original annotations and an additional rater, as well as those between original annotations and model predictions, was assessed using the paired Student’s *t*-test. Similarly, paired Student’s *t*-tests were applied to compare different models, with multiple comparisons adjusted using the Li correction^70^. *F*-tests were used to evaluate ICC significance^71^. Homogeneity of variances was tested using Levene’s test, and normality was verified using the Shapiro-Wilk test. Whenever these assumptions were violated, we instead applied the nonparametric Wilcoxon signed-rank test. The significance level for all statistical tests was set at 0.05.

### Ethics approval and consent to participate

All research involving human participants was performed in accordance with the Declaration of Helsinki. Written informed consent was obtained from all participants for the original studies. Detailed ethics approvals for each contributing cohort are listed in Methods 4.1. The user-centered design study evaluating the AID-FOG platform received separate approval from the Social and Societal Ethics Committee of KU Leuven (G-2023-7462), and all participating experts provided written informed consent.

## Supplementary information


Supplementary information
Supplementary Video 1


## Data Availability

The local cohort datasets in KU Leuven and the external Radboud dataset analyzed in this study are not publicly available due to restrictions on sharing sensitive patient health information. In contrast, the following external datasets are publicly accessible: • Stanford^34^: Available at https://github.com/stanfordnmbl/imu-fog-detection • Twente^36,37^: Available at https://data.4tu.nl/datasets/40e06061-f441-43b5-9235-006829206509 • Federal^35^: Available at https://figshare.com/articles/dataset/14984667 • Xuanwu^38^: Available at https://data.mendeley.com/datasets/r8gmbtv7w2/3

## Code availability

The DL architectures were implemented in PyTorch 2.2.2+cu118^72^, leveraging publicly available repositories for MS-TCN^28^, ASFormer^29^, and LTContext^30^. Statistical analyses and visualizations were conducted using SciPy 1.7.11, bioinfokit 2.1.0, statsmodels 0.13.2, and pingouin 0.3.12 for statistical computations, and matplotlib 3.8.4 for plotting. The scripts were written in Python 3.10.14, with scamamp^73^ implemented in R for additional statistical modeling. The code developed for this study is not publicly available, but may be provided upon reasonable request.
