# Supplementary material for: Deep learning for freezing of gait assessment using inertial measurement units: a multicentre validation study
Source: NPJ Parkinsons Dis. 2026 Jun 2;12:209. doi: 10.1038/s41531-026-01407-7 (PMC13530201; doi:10.1038/s41531-026-01407-7)
Supplement: Supplementary file 1 [file 41531_2026_1407_MOESM1_ESM.pdf]

Supplementary Information

Hyperparameter search results

Hyperparameter values for the three architectures (MS-TCN, ASFormer, LTContext) were tuned on the KU Leuven cohort using the sequential procedure and selection criteria described in Methods 4.6. Supplementary Tables 1-4 summarizes the overall performance of models trained with different hyperparameter settings, indicating minimal performance variation across configurations. Following the selection criteria outlined earlier, the final hyperparameter configurations for all three models are presented in Supplementary Table 5.

Model	Batch size	KUL F1 mean	F1 p-value	F1@50 mean	F1@50 p-value	#FPs per trial mean	#FPs per trial p-value	FP duration per trial mean	FP duration per trial p-value	ICC %TF	ICC #FOG
LTContext	1*	0.266	0.976	0.116	0.842	**0.201**	0.312	**0.100**	0.241	**0.870**	0.587
	2	0.273		0.111		0.335		0.259		0.866	0.599
	4	0.276		0.119		0.317		0.297		0.845	**0.658**
	8	0.258		0.099		0.434		0.345		0.777	0.588
ASFormer	1*	**0.361**	-	**0.144**	-	**1.324**	-	**0.400**	-	**0.813**	**0.661**
MS-TCN	1*	0.385	0.975	**0.235**	0.920	**0.513**	0.683	0.196	0.156	**0.921**	**0.805**
	2	0.375		0.214		0.788		0.246		0.905	0.784
	4	0.385		0.216		0.948		**0.192**		0.905	0.804
	8	**0.404**		0.234		0.875		0.297		0.895	0.787
	16	0.389		0.211		1.031		0.319		0.881	0.796

Supplementary Table 1 Impact of batch size on overall performance

This table illustrates the impact of batch size on overall performance for all three models. Note that for ASFormer, only a batch size of 1 is supported as per the original study [1]. Since all p-values exceed 0.05, the configuration with the highest ICC(%TF) was selected, denoted by an asterisk (*); i.e., a batch size of 1 for all three models.

Model	#Layers	KUL F1 mean	F1 p-value	F1@50 mean	F1@50 p-value	#FPs per trial mean	#FPs per trial p-value	FP duration per trial mean	FP duration per trial p-value	ICC %TF	ICC #FOG
LTContext	6	**0.323**	0.53	**0.149**	0.541	0.525	0.181	0.352	0.186	0.794	0.582
	8	0.310		0.145		0.566		0.267		0.841	0.641
	10*	0.266		0.116		**0.201**		**0.100**		**0.870**	0.587
	12	0.289		0.123		0.575		0.266		0.867	**0.725**
ASFormer	4*	0.396	0.612	**0.237**	**0.005**	**0.646**	**0.048**	**0.190**	**0.011**	0.846	0.755
	6	**0.411**		0.220		1.024		0.350		**0.868**	**0.825**
	8	0.376		0.161		1.324		0.329		0.825	0.712
	10	0.361		0.144		1.324		0.400		0.813	0.661
MS-TCN	6	**0.403**	0.961	0.232	0.995	0.508	0.571	0.263	0.605	0.901	**0.829**
	8	0.389		0.228		0.685		0.267		0.894	0.746
	10	0.385		**0.235**		0.513		**0.196**		0.921	0.805
	12*	0.382		0.227		**0.456**		0.197		**0.927**	0.806

Supplementary Table 2 Impact of #layers on overall performance

This table shows the impact of number of layers on performance. For LTContext and MS-TCN, all p-values exceeded 0.05; thus, the configuration with the highest ICC(%TF) (marked with *) was chosen. For ASFormer, significant differences in F1@50, #FP per trial, and FP duration per trial led to selecting the model with 4 layers.

Model	#Stages	KUL									
		F1		F1@50		#FPs per trial		FP duration per trial		ICC	
		mean	p-value	mean	p-value	mean	p-value	mean	p-value	%TF	#FOG
LTContext	3	0.266		0.116		**0.201**		**0.100**		0.870	**0.587**
	4*	0.291	0.693	0.123	0.593	0.302	0.362	0.230	0.232	**0.886**	0.573
	5	**0.312**		**0.142**		0.353		0.294		0.871	0.505
ASFormer	2	**0.408**	0.308	**0.289**	0.911	1.223	**< 0.001**	0.261	**0.043**	0.817	0.748
	3*	0.396		0.237		**0.646**		**0.190**		**0.846**	**0.755**
MS-TCN	3	**0.389**		**0.243**		0.643		0.200		0.903	**0.810**
	4*	0.382	0.973	0.227	0.658	**0.456**	0.490	**0.197**	0.606	**0.927**	0.806
	5	0.380		0.214		0.674		0.258		0.905	0.804

Supplementary Table 3 Impact of #stages on overall performance

This table shows the effect of stage number on performance. For LTContext and MS-TCN, since all p-values were > 0.05, the configuration with the highest ICC(%TF) was chosen (marked with *). For ASFormer, the 3-stage configuration achieved the highest ICC for both %TF and #FOG, with significant differences in #FP per trial and FP duration per trial. Note that the 4-stage ASFormer model was not evaluated due to an out-of-memory error.

Model	#Features	KUL									
		F1		F1@50		#FPs per trial		FP duration per trial		ICC	
		mean	p-value	mean	p-value	mean	p-value	mean	p-value	%TF	#FOG
LTContext	32	0.263		0.092		0.445		0.479		0.823	0.437
	64*	**0.291**	0.050	**0.123**	0.228	**0.302**	0.675	0.230	**0.018**	**0.886**	**0.573**
	128	0.193		0.085		0.695		**0.133**		0.690	0.520
ASFormer	32	0.312		0.157		**0.455**		**0.142**		0.832	**0.833**
	64	0.396	0.060	**0.237**	0.075	0.646	0.056	0.190	0.356	0.846	0.755
	128*	0.396		0.220		0.860		0.311		**0.866**	0.769
	256	**0.416**		0.226		0.975		0.242		0.843	0.714
MS-TCN	32	**0.396**		0.211		0.876		0.288		0.876	0.660
	64	0.382		**0.227**		0.456		0.197		0.927	0.806
	128	0.370	0.952	0.224	0.932	0.456	0.108	0.136	**0.007**	0.940	0.842
	256*	0.365		0.219		**0.420**		0.126		**0.942**	**0.901**
	512	0.368		0.201		0.817		**0.104**		0.931	0.858

Supplementary Table 4 Impact of #features on overall performance

This table illustrates how varying the number of hidden features affects the overall performance of all three models. When a p-value is below 0.05, indicating a statistically significant difference, the best performing model is highlighted in bold, and any underlined number indicates a significant deviation from that best performer. Based on our selection criteria, the optimal configurations were identified as follows: MS-TCN with 256 features, ASFormer with 128 features, and LTContext with 64 features.

Model	Batch size	#Layers	#Stages	#Features
LTContext	1	10	4	64
ASFormer	1	4	3	128
MS-TCN	1	6	4	256

Supplementary Table 5 Selected hyperparameters for the three models

Architecture comparison results

Per-cohort performance of MS-TCN, ASFormer, and LTContext on the six external datasets is reported in Supplementary Table 6, complementing the cross-cohort means shown in Table 2 of the main text.

As shown in Supplementary Table 6, no significant differences were observed in F1 and F1@50 among the three models. LTContext demonstrated a significantly lower #FP and FP duration per trial compared to MS-TCN and significantly lower #FP per trial than ASFormer. Additionally, LTContext recorded the highest ICC values

for both %TF and #FOG. Based on these results, LTContext was selected for further experiments.

Convergence analysis for fine-tuning step count

To determine an appropriate fixed number of fine-tuning steps for the cohort adaptation experiments described in Methods 4.6, we analysed convergence behaviour on the largest available training condition (360 minutes) across each external cohort, using 7-fold participant-level cross-validation with early stopping (patience of five epochs).

Our analysis indicated that across the 7-fold cross-validation, the model required an average of 2920.71 ± 1050.34 steps to converge. Therefore, we selected a fixed number of 3000 steps for all fine-tuning procedures, providing a consistent and sufficient training schedule across all dataset sizes.

Model and third-rater annotations on the Mobilise-D Cohort

Supplementary Figures 1 and 2 present 29 trials from the Mobilise-D cohort, annotated by a third rater at KU Leuven with prior experience in FOG annotation. The rater was blinded to the fact that all provided trials contained FOG episodes previously annotated as ground truth by another expert. As shown in the figures, FOG episodes in this dataset are extremely short, mostly lasting less than half a second. The original annotating expert also noted that in some cases, FOG was observed on only a single leg.

The figures illustrate that after fine-tuning, the model aligned more closely with the ground truth annotations than with the third rater, as fine-tuning was performed based on the original expert's annotations. The ICC(%TF) and ICC(#FOG) between the fine-tuned model and the ground truth were 0.540 and 0.756, respectively, whereas the ICC between the third rater and the ground truth was 0.757 and 0.543. These results indicate that FOG annotation in this dataset presents challenges even for human raters and that, after fine-tuning, the model demonstrated a level of agreement comparable to that of an additional human annotator.

Model	External cohorts (ICC)												Arithmetic averages									
	Stanford		Mobilise-D		Radboud		Federal		Twente		Xuanwu		F1		F1@50		#FP		FP-Duration		ICC	
	%TF	#FOG	%TF	#FOG	%TF	#FOG	%TF	#FOG	%TF	#FOG	%TF	#FOG	mean	p-value	mean	p-value	mean	p-value	mean	p-value	%TF	#FOG
LTContext*	**0.616**	**0.556**	0.426	0.291	0.481	0.098	**0.417**	0.001	0.610	**0.095**	0.824	**0.748**	**0.390**		**0.185**		**1.690**		5.076		**0.562**	**0.298**
ASFormer	0.059	0.011	0.451	0.327	**0.482**	0.081	0.361	**0.020**	**0.862**	0.034	0.750	0.326	0.356	0.982	0.109	0.563	5.151	0.485	**0.635**	0.193	0.494	0.133
MS-TCN	0.287	0.039	**0.582**	**0.444**	0.290	**0.115**	0.145	-0.012	0.472	0.036	**0.850**	0.449	0.340		0.146		3.937		1.749		0.438	0.179

Supplementary Table 6 Model selection results

This table compares overall performance metrics: sample-wise F1 score, segment-wise F1@50, mean #FP per trial, FP duration per trial, and ICC for %TF and #FOG, with detailed ICC values provided for each external cohort. When a p-value is below 0.05, indicating a statistically significant difference, the best performing model is highlighted in bold, and any underlined number indicates a significant deviation from that best performer. Based on these results, LTContext was selected for further experiments.

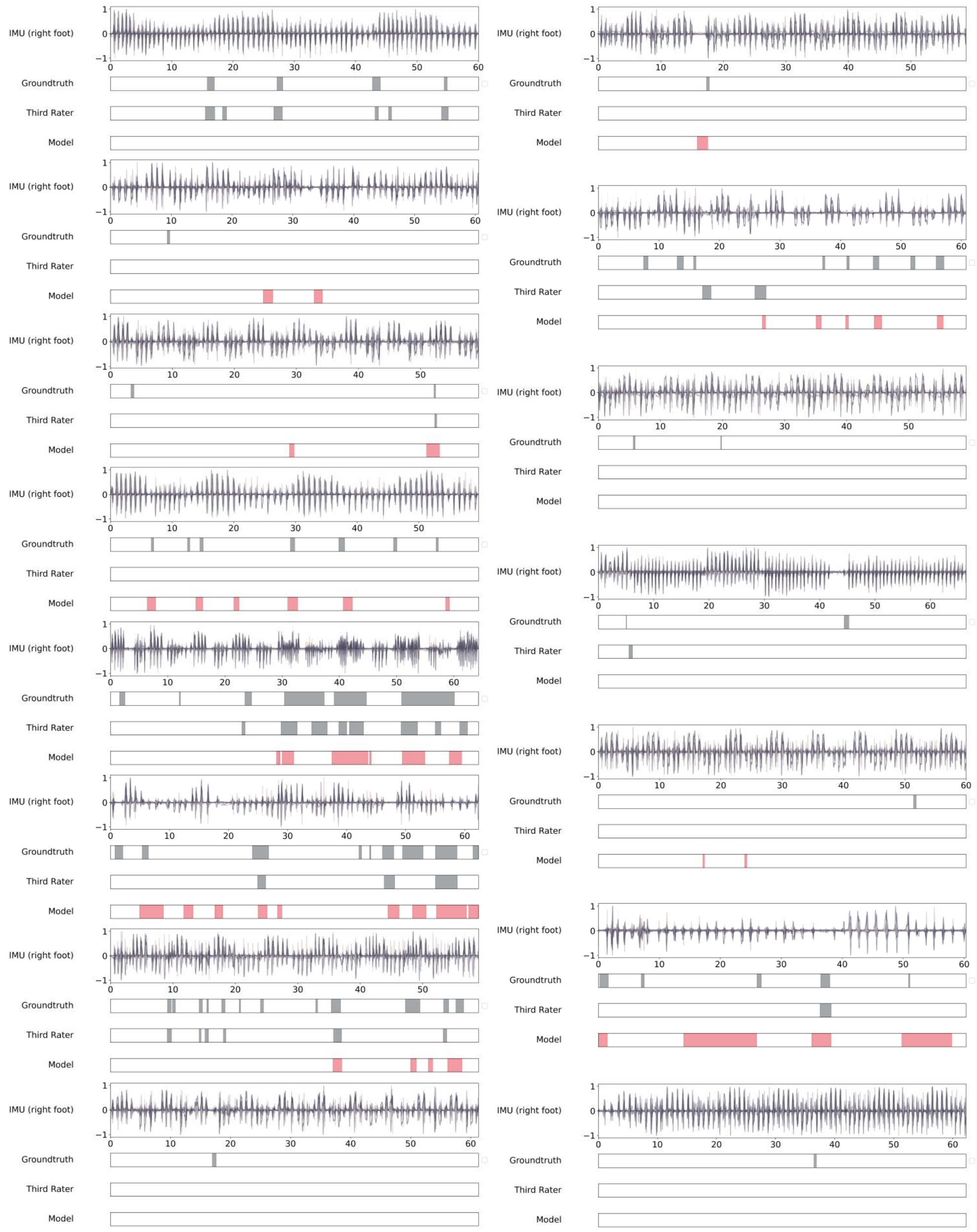

Supplementary Figure 1 Model (fine-tuned with 50 minute data) predictions versus expert and third-rater annotations for the first 15 Mobilise-D trials. The figure shows 15 trials from the Mobilise-D cohort annotated by a third independent rater at KU Leuven. For each trial, four rows are shown from top to bottom: the normalized right-foot IMU signal (amplitude between -1 and 1) plotted against time in seconds; the ground-truth annotation from the original Mobilise-D expert (gray horizontal bars); the annotation produced by the third rater at KU Leuven (gray horizontal bars, second row); and the model predictions after fine-tuning with 50 minutes of Mobilise-D data (red horizontal bars). Abbreviations: FOG, freezing of gait; IMU, inertial measurement unit.

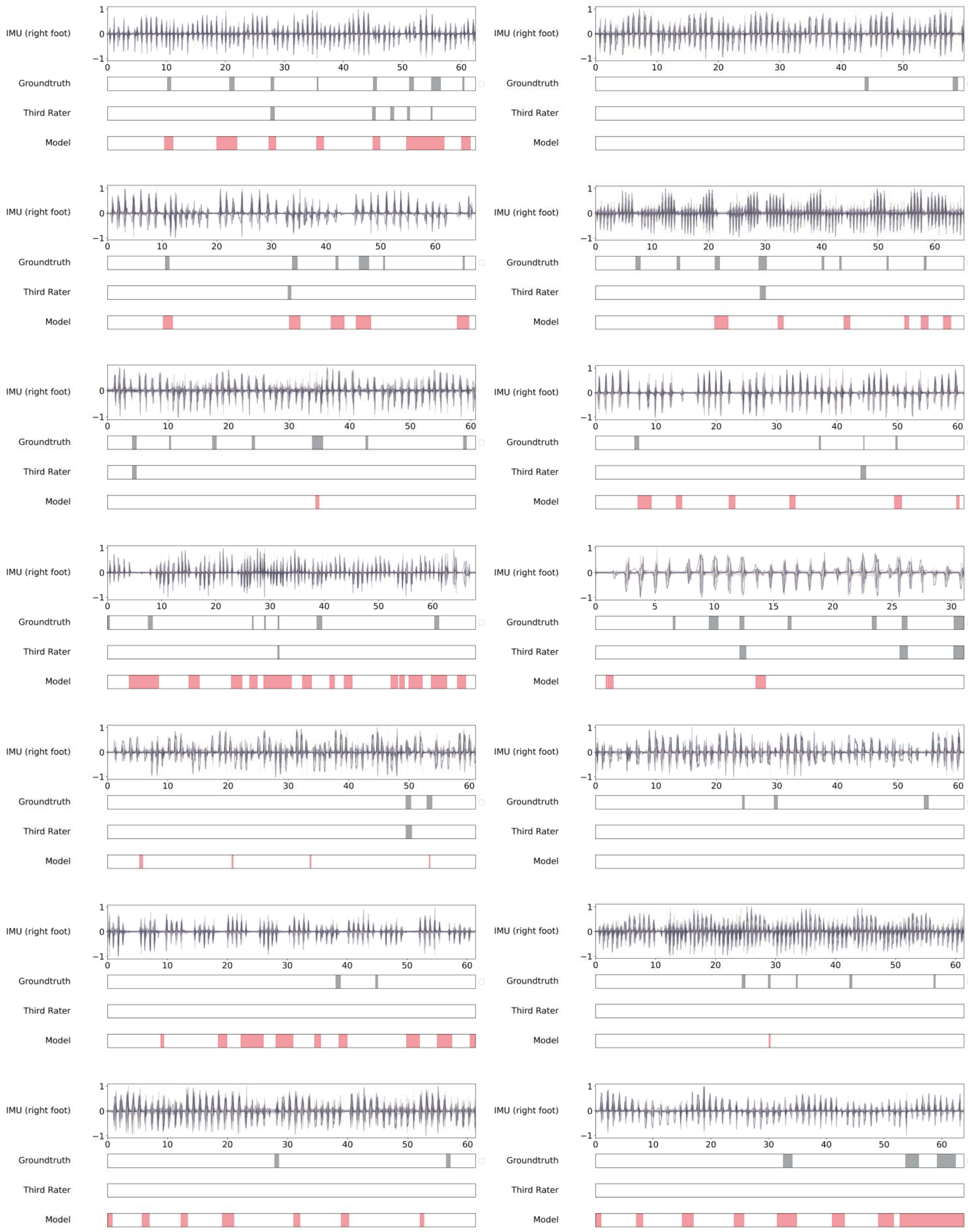

Supplementary Figure 2 Model (fine-tuned with 50 minute data) predictions versus expert and third-rater annotations for Mobilise-D. The figure shows the remaining 14 trials from the Mobilise-D cohort annotated by a third independent rater at KU Leuven. For each trial, four rows are shown from top to bottom: the normalized right-foot IMU signal (amplitude between -1 and 1) plotted against time in seconds; the ground-truth annotation from the original Mobilise-D expert (gray horizontal bars); the annotation produced by the third rater at KU Leuven (gray horizontal bars, second row); and the model predictions after fine-tuning with 90 minutes of Mobilise-D data (red horizontal bars). Abbreviations: FOG, freezing of gait; IMU, inertial measurement unit.

Dataset-level model performance during fine-tuning

Supplementary Figure 4 illustrates the ICC(%TF) trends across the six external datasets for models trained from scratch, pre-trained, and fine-tuned. Fine-tuning improved ICC(%TF) in four out of six cohorts (Stanford, Radboud, Federal, and Twente), while performance in Mobilise-D and Xuanwu remained comparable to the pre-trained baseline.

A closer inspection of sample-wise F1 scores and mean FP duration per trial reveals the underlying factors. Fine-tuning substantially reduced FP duration in non-FOG trials for Radboud and Twente and moderately for Federal and Xuanwu. In contrast, FP duration increased in Stanford and Mobilise-D. However, Stanford also showed an improvement in sample-wise F1, while Mobilise-D's F1 remained largely unchanged.

These findings suggest that improvements in ICC(%TF) stem from either better detection of FOG in FOG trials (higher F1) or fewer FPs in non-FOG trials (shorter FP durations). Specifically, fine-tuning enhanced detection in Stanford and effectively suppressed FPs in Radboud, Federal, and Twente. In Xuanwu, fine-tuning preserved FOG detection performance and slightly reduced FPs, yielding a modest improvement in ICC(%TF). In Mobilise-D, however, fine-tuning led to increased FPs and predicted FOG episodes, limiting ICC gains. Overall, fine-tuning had the strongest positive effect on Radboud, Federal, and Twente, a moderate effect on Stanford and Xuanwu, and minimal impact on Mobilise-D. The lower average ICC(%TF) was primarily driven by Mobilise-D's limited improvement. While FOG episodes in Mobilise-D are substantially shorter on average (mean 0.98 s) than in KU Leuven (mean 3.19 s), Supplementary Figure 3 shows that the KU Leuven training data also contains short episodes and exhibits a similarly right-skewed duration distribution. This overlap suggests that the limited generalization to Mobilise-D is not solely attributable to a lack of short episodes in the training data, but rather reflects broader differences in patient spectrum: the Mobilise-D cohort predominantly comprises mild freezers assessed during 360 degree turning-in-place task, a clinical context that differs markedly from the more severe freezer profiles in the KU Leuven training cohort. These patient spectrum differences, combined with the inherent difficulty of annotating very brief FOG episodes even for experienced human raters (see Supplementary Figuress 1 and 2), likely contribute to the persistently lower ICC observed for this cohort.

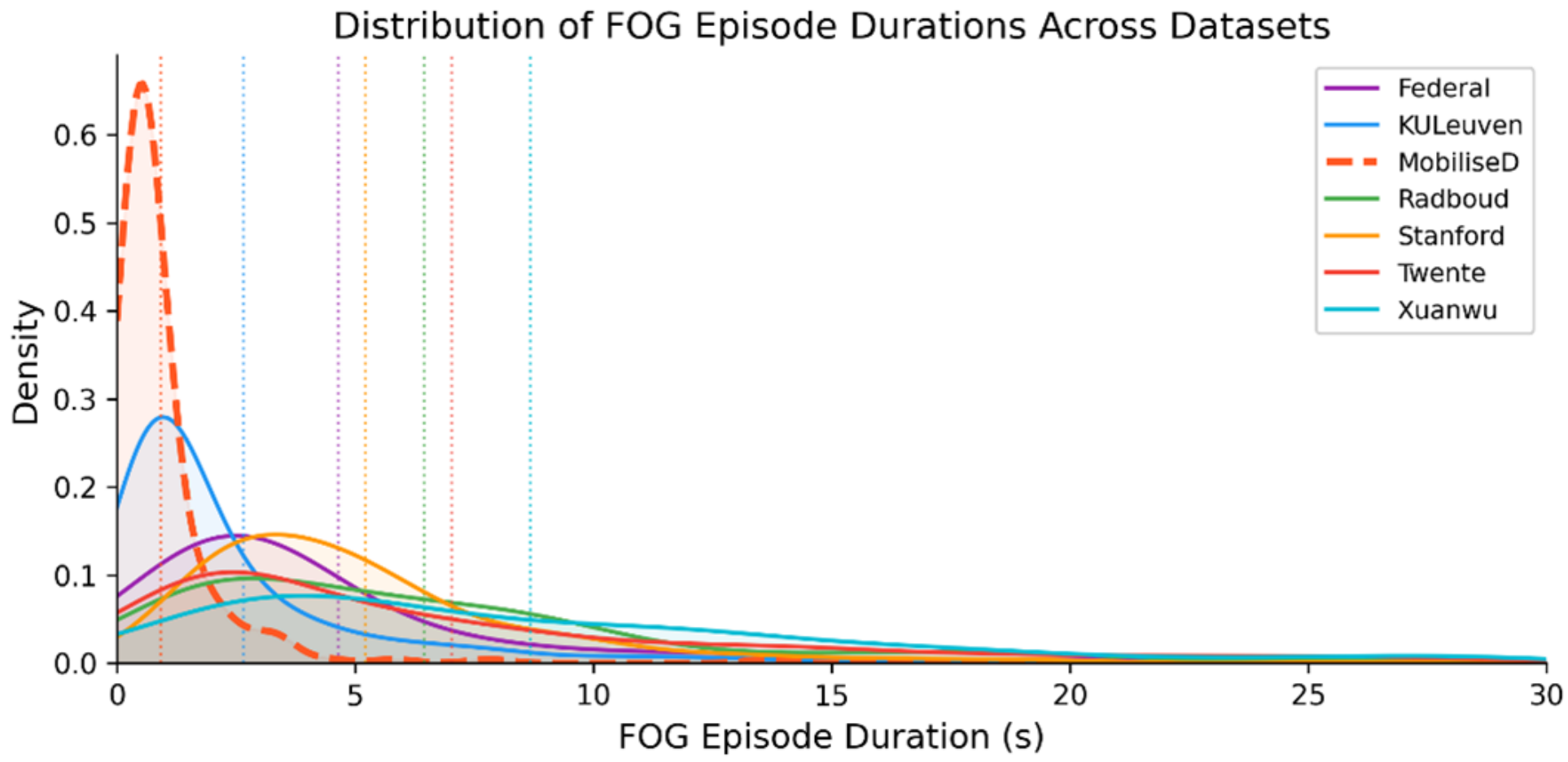

Supplementary Figure 3 Distribution of freezing of gait episode durations across the seven cohorts. The figure shows the probability density of FOG episode duration (horizontal axis, in seconds, from 0 to 30) for each of the seven cohorts, plotted as kernel density estimates. Each cohort is represented by a distinct line color, with the legend identifying: Federal (purple), KU Leuven (blue), Mobilise-D (red dashed), Radboud (green), Stanford (yellow), Twente (red), and Xuanwu (cyan). The vertical dashed lines in matching colors indicate the mean episode duration for each cohort. Mobilise-D shows the highest density at very short durations (<1 s), reflecting its predominance of mild freezers, whereas other cohorts exhibit broader, right-skewed distributions. Abbreviations: FOG, freezing of gait.

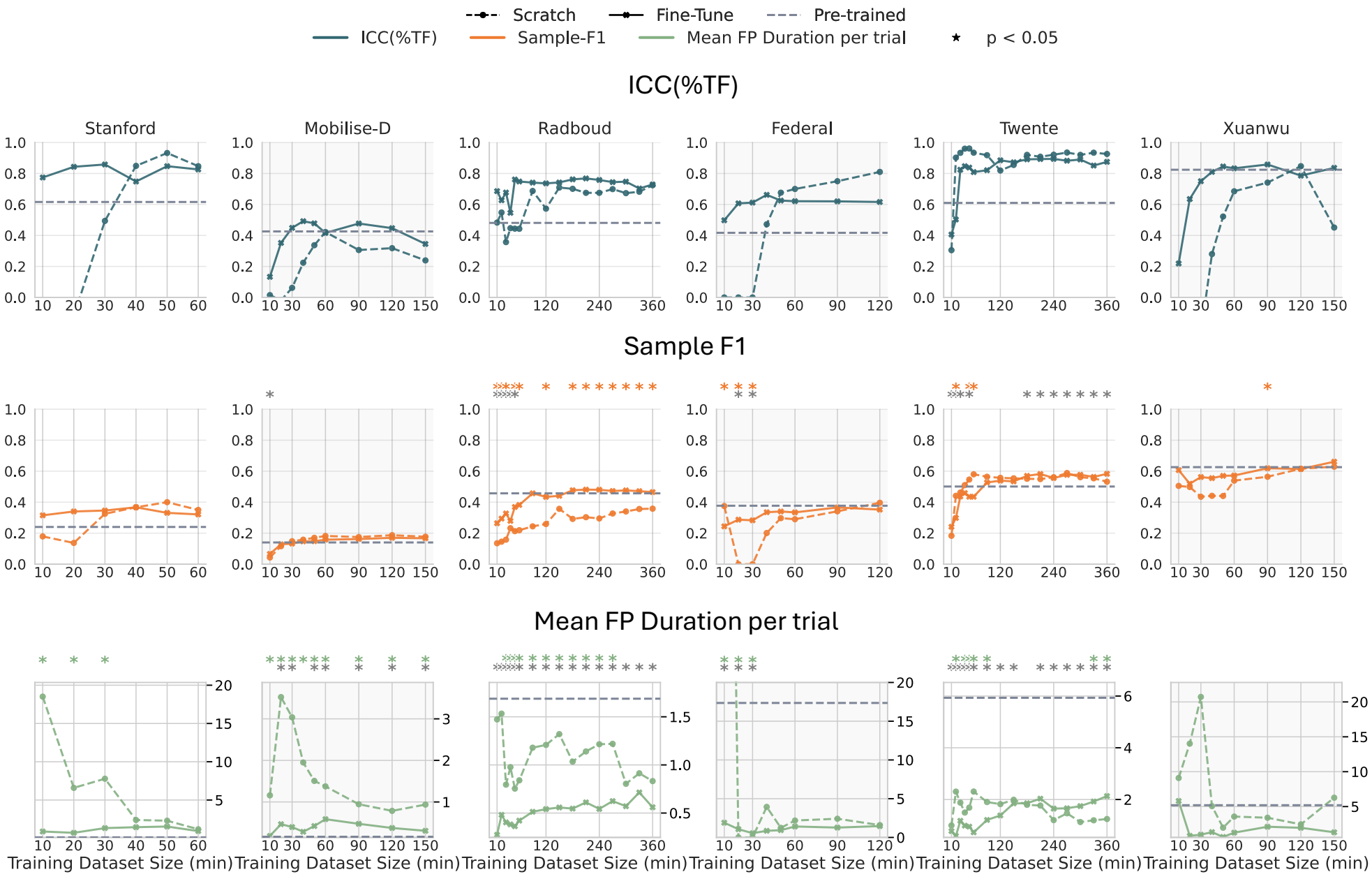

Supplementary Figure 4 Dataset-level model performance during fine-tuning for percentage of time spent frozen and supporting metrics. The figure presents three rows of six panels each, one column per external cohort (Stanford, Mobilise-D, Radboud, Federal, Twente, and Xuanwu), as a function of training dataset size in minutes. a) Top row: intraclass correlation coefficient for percentage of time spent frozen (ICC(%TF)). b) Middle row: sample-wise F1 score on FOG trials. c) Bottom row: mean false-positive duration per non-FOG trial, in seconds. In each panel, three training conditions are compared: models trained from scratch on the external cohort are shown as a colored dashed line with circle markers; the KU Leuven-pretrained model evaluated without adaptation is shown as a gray dashed horizontal line; and the same pretrained model after fine-tuning on the external cohort is shown as a colored solid line with circle markers. The colors of the dashed (scratch) and solid (fine-tuned) lines match within each panel to allow direct comparison between the two adaptation strategies on the same cohort. Gray asterisks above each panel denote training durations at which the fine-tuned model differs significantly ($p < 0.05$) from the pre-trained baseline, and colored asterisks denote training durations at which the fine-tuned model differs significantly from the model trained from scratch. Abbreviations: ICC, intraclass correlation coefficient; %TF, percentage of time spent frozen; FP, false positive; FOG, freezing of gait.

Supplementary Figure 5 shows ICC(#FOG) scores. Fine-tuning improved performance in four datasets (Radboud, Federal, Twente, and Xuanwu), while results for Stanford and Mobilise-D were similar to the pre-trained baseline.

Analysis of F1@50 and average #FPs per trial provides insight into these patterns. Fine-tuning significantly reduced #FPs in Radboud, Federal, and Twente, and not significantly in Xuanwu. F1@50 remained largely stable compared to the pre-trained model, except in Twente, where it significantly improved after incorporating more than 50 minutes of data. In Stanford, fine-tuning slightly increased both F1@50 and #FPs, though the changes were not statistically significant. In Mobilise-D, fine-tuning initially caused a significant rise in #FPs, while F1@50 remained unchanged.

These results suggest that ICC(#FOG) improvements are driven by either better segment-wise FOG detection (higher F1@50) or fewer FPs in non-FOG trials. Fine-tuning was especially effective in reducing FPs for Radboud, Federal, Twente, and Xuanwu, leading to higher ICC(#FOG). In contrast, Stanford and Mobilise-D showed minimal gains, with Mobilise-D eventually improving after extended fine-tuning, while Stanford showed no such trend.

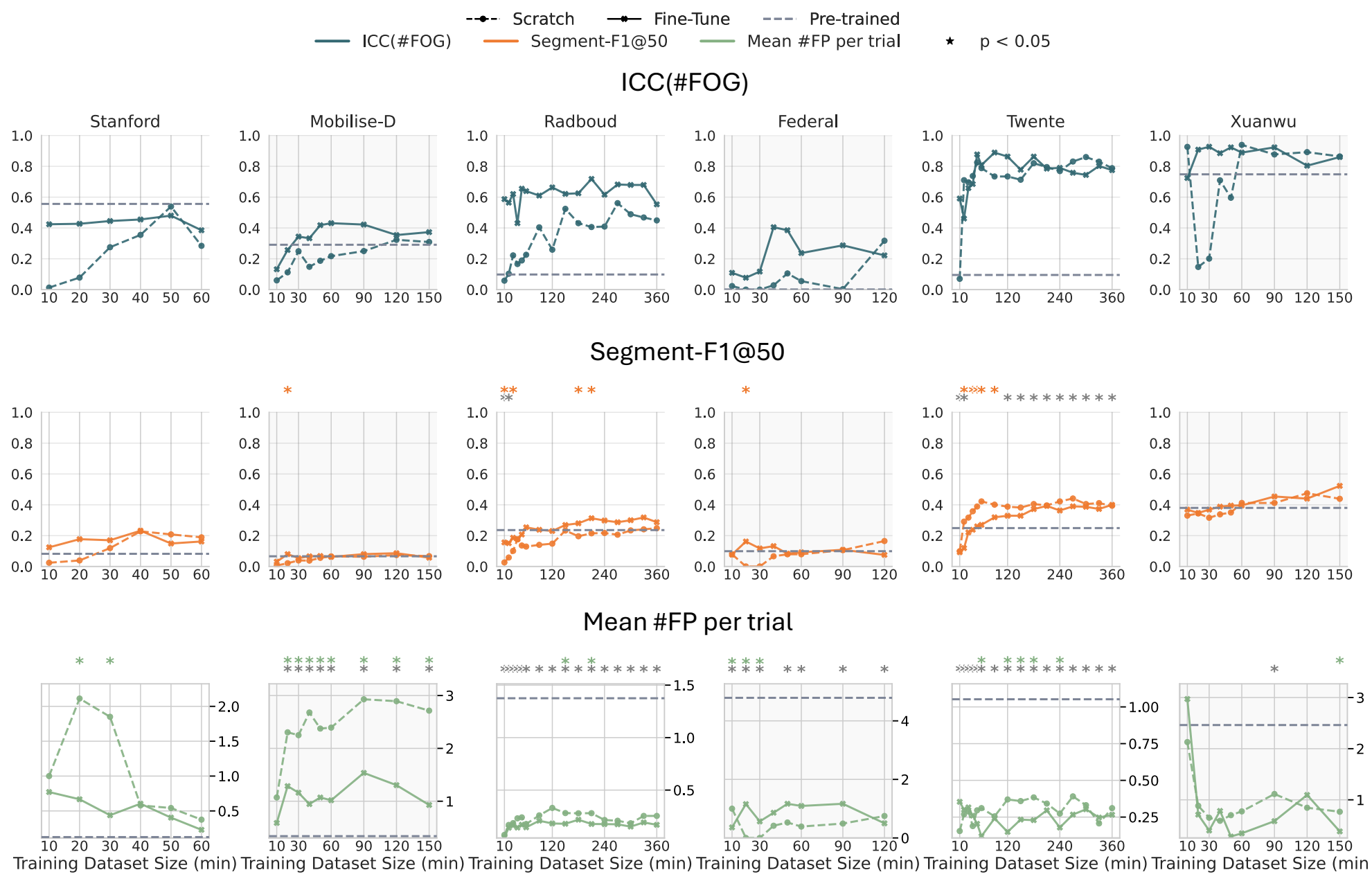

Supplementary Figure 5 Dataset-level model performance during fine-tuning for the number of freezing of gait episodes and supporting metrics. The figure presents three rows of six panels each, one column per external cohort (Stanford, Mobilise-D, Radboud, Federal, Twente, and Xuanwu), as a function of training dataset size in minutes. a) Top row: intraclass correlation coefficient for the number of FOG episodes (ICC(#FOG)). b) Middle row: segment-wise F1@50 score on FOG trials. c) Bottom row: mean number of false-positive episodes per non-FOG trial (#FP per trial). In each panel, three training conditions are compared: models trained from scratch on the external cohort are shown as a colored dashed line with circle markers; the KU Leuven-pretrained model evaluated without adaptation is shown as a gray dashed horizontal line; and the same pretrained model after fine-tuning on the external cohort is shown as a colored solid line with circle markers. The colors of the dashed (scratch) and solid (fine-tuned) lines match within each panel to allow direct comparison between the two adaptation strategies on the same cohort. Gray asterisks above each panel denote training durations at which the fine-tuned model differs significantly ($p < 0.05$) from the pre-trained baseline, and colored asterisks denote training durations at which the fine-tuned model differs significantly from the model trained from scratch. Abbreviations: ICC, intraclass correlation coefficient; #FOG, number of FOG episodes; #FP, number of false-positive episodes; FOG, freezing of gait.

Supplementary evaluation metrics for pre-trained and fine-tuned models

To facilitate comparison with prior FOG detection work [2–6] that typically reports sample- or window-level metrics pooled across the entire dataset, we additionally computed the conventional metrics (accuracy, recall, specificity, precision, F1, and area under the receiver operating characteristic curve (AUROC)) by pooling all samples across each cohort, complementing the trial-averaged metrics reported in the main text. Supplementary Table 7 reports these metrics for the KU Leuven internal cohort and for each external cohort under the pre-trained and fine-tuned (50 minutes) conditions.

As summarized in Supplementary Table 7, fine-tuning LTContext on 50 minutes of cohort-specific annotations yields a 6.4 % increase in accuracy, 13.9 % in specificity and 6.2 % in F1 score, while with a 17.5 % drop in recall and 0.9 % in AUROC relative to the KU Leuven–pretrained baseline model. Here, specificity quantifies the model's ability to avoid FP detections; recall measures the proportion of actual FOG episodes correctly identified; precision indicates the fraction of predicted FOG events that are true; and the F1 score harmonizes precision and recall into a single performance metric. Consequently, if the primary objective is to minimize missed FOG events (i.e. false negatives), the pretrained model, demonstrating the highest recall in four of six cohorts, remains the preferred choice. However, its comparatively low precision indicates that many of the detected episodes may be FPs, which could burden clinicians with unnecessary adjustments. In contrast, when the priority is to reduce false alarms, the fine-tuned model, achieving higher specificity and precision in four of six cohorts, should be employed.

AID-FOG platform development

The AID-FOG platform is a proof-of-concept web application that supports researchers in reviewing and refining FOG annotations generated by the deep-learning algorithm. The platform was developed in collaboration with three clinical experts at KU Leuven, each with over two years of experience in FOG annotation (averaging nine years specifically in FOG labelling), and evolved across three successive versions. Version 1.0 contained the core requirements for an online FOG annotation platform, including basic interactive annotation functions and visualization of IMU signals and videos. Version 2.1 added improved annotation adjustment, support for adding notes on annotations, FOG severity calculation and display (#FOG, %TF), upload and download of annotations in comma-separated-values format, and user authentication. Version 2.2 added keyboard shortcuts (e.g., Shift + Drag, Ctrl+Z and Ctrl+Y for undo and redo), support for detailed labelling of different FOG manifestation and movement-cessation types, a project-management file system, and the option to download annotations, videos, and CSV files as a single archive. Supplementary Figure 6 illustrates the feature evolution across the three versions, and Supplementary Figure 7 shows the FOG annotation page of the current version. Each version was rated by the three experts using the IBM Computer System Usability Questionnaire (CSUQ) [7], a

	Accuracy		Recall		Specificity		Precision		F1		AUROC	
**Cohort**	Value	Δ%	Value	Δ%	Value	Δ%	Value	Δ%	Value	Δ%	Value	Δ%
KU Leuven												
Internal	0.880		0.857		0.884		0.546		0.667		0.871	
Stanford												
Pre-trained	0.805		0.358		0.959		0.753		0.486		0.659	
Tuned (50 min)	0.810	+0.6 %	0.531	+48.3 %	0.906	-5.5 %	0.661	-12.2 %	0.589	+21.2 %	0.719	+9.1 %
Mobilise-D												
Pre-trained	0.946		0.312		0.973		0.324		0.318		0.642	
Tuned (50 min)	0.918	-3.0 %	0.464	+48.7 %	0.937	-3.7 %	0.236	-27.2 %	0.313	-1.6 %	0.700	+9.0 %
Federal												
Pre-trained	0.670		0.928		0.610		0.357		0.516		0.769	
Tuned (50 min)	0.836	+24.8 %	0.651	-29.8 %	0.880	+44.3 %	0.558	+56.3 %	0.601	+16.5 %	0.765	-0.5 %
Radboud												
Pre-trained	0.830		0.796		0.833		0.298		0.434		0.814	
Tuned (50 min)	0.906	+9.2 %	0.522	-34.4 %	0.941	+13.0 %	0.439	+47.3 %	0.477	+9.9 %	0.731	-10.2 %
Twente												
Pre-trained	0.760		0.952		0.699		0.503		0.659		0.825	
Tuned (50 min)	0.860	+13.2 %	0.661	-30.6 %	0.923	+32.1 %	0.734	+45.9 %	0.696	+5.6 %	0.792	-4.0 %
Xuanwu												
Pre-trained	0.790		0.880		0.720		0.712		0.787		0.800	
Tuned (50 min)	0.778	-1.5 %	0.656	-25.5 %	0.874	+21.4 %	0.804	+12.9 %	0.722	-8.3 %	0.765	-4.4 %
**Averaged over six external cohorts**												
Pre-trained	0.800		0.704		0.799		0.491		0.533		0.752	
Tuned (50 min)	0.851	+6.4 %	0.581	-17.5 %	0.910	+13.9 %	0.572	+16.5 %	0.566	+6.2 %	0.745	-0.9 %

Supplementary Table 7 Performance metrics across six external cohorts

This table presents the sample-wise performance metrics, i.e., accuracy, recall, specificity, precision, F1 score, and AUROC, computed over the entire dataset for each external cohort.

19-item Likert-scale instrument that produces four usability scores: system usefulness, information quality, interface quality, and overall usability. In the third iteration, the experts also rated the widely used ELAN software [8, 9] using the same instrument as a reference comparison. Ethical approval for the user-centred design work was obtained from the Social and Societal Ethics Committee of KU Leuven (G-2023-7462), and all participating experts provided written informed consent.

The current version of the AID-FOG platform

The currently developed version of the AID-FOG platform enables human raters to upload synchronized IMU and video data and view model predictions derived from IMU signals alongside the IMU data and video. Annotations can be reviewed, adjusted, and downloaded as the commonly used ELAN software [8]. In addition, users can upload corrected annotations to the backend, potentially enabling model fine-tuning. Supplementary Figure 7 illustrates the FOG annotation page in the AID-FOG platform, Supplementary Video 1 provides a live demonstration of its main functionalities, and the current version of the platform is accessible at aidfog.be.

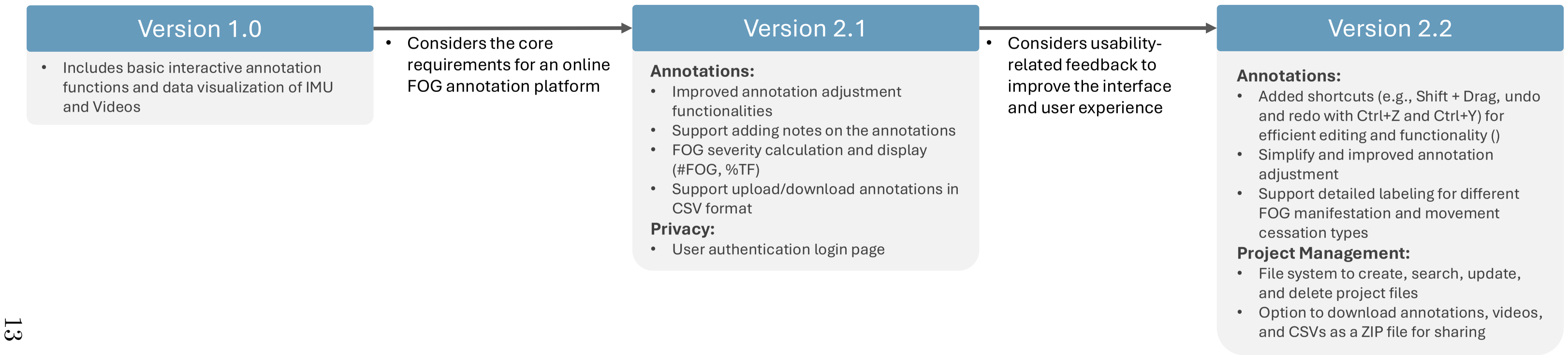

Supplementary Figure 6 Feature evolution of the AID-FOG platform across three design iterations. The figure shows the successive versions of the AID-FOG web platform and the feature categories added at each iteration, based on feedback from three internal clinical experts at KU Leuven. Version 1.0 establishes the core requirements for an online FOG annotation platform, including basic interactive annotation functions and data visualization of IMU signals and videos. Version 2.1 incorporates usability-related feedback to improve the interface and user experience: improved annotation adjustment functionalities, support for adding notes on annotations, FOG severity calculation and display (#FOG, %TF), support for upload and download of annotations in comma-separated-values format, and user authentication via a login page. Version 2.2 refines annotations with keyboard shortcuts (e.g., Shift + Drag, undo and redo with Ctrl+Z and Ctrl+Y), simplified annotation adjustment, support for detailed labeling of different FOG manifestation and movement-cessation types, a project-management file system to create, search, update, and delete project files, and a download option for annotations, videos, and comma-separated-values files as a single archive file. Abbreviations: AID-FOG, AI-Driven Freezing of Gait; FOG, freezing of gait; IMU, inertial measurement unit; %TF, percentage of time spent frozen; #FOG, number of FOG episodes.

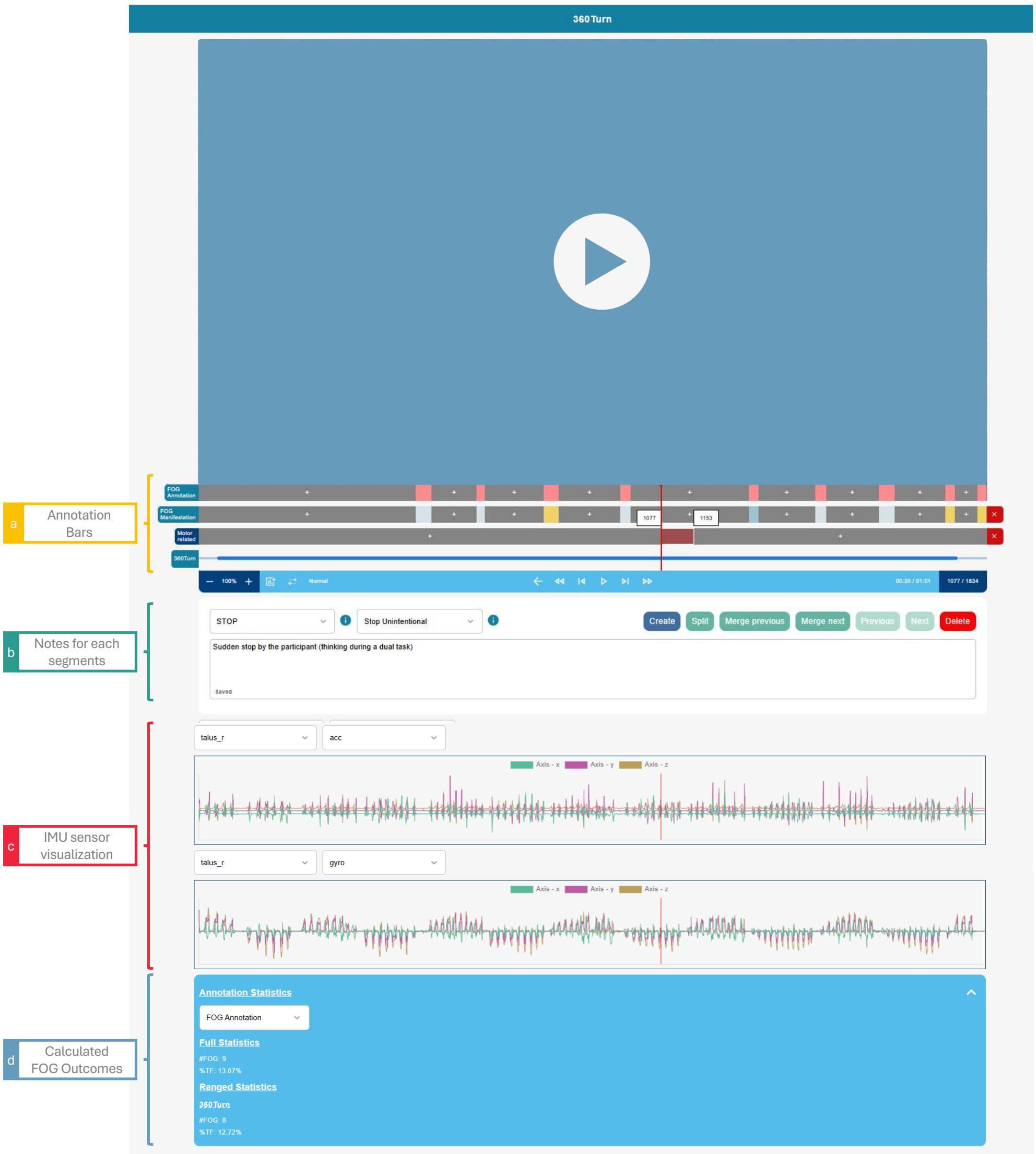

Supplementary Figure 7 Overview of the freezing of gait annotation page on the AID-FOG platform. The figure shows a screenshot of the main annotation interface of the AID-FOG web application. The video player is shown at the top, and four key panels are displayed below. a) Annotation bars indicating freezing of gait (FOG) and task segments, initially populated by model predictions on first access and adjustable by the user. b) A notes field allowing the rater to mark specific events associated with each annotated segment. c) Synchronized IMU sensor data uploaded by the user, displayed as three-axis traces and used by the backend to generate model predictions. d) Calculated FOG outcome metrics, including percentage of time spent frozen (%TF) and the number of FOG episodes (#FOG), reported for both the currently annotated task and the entire trial. Abbreviations: AID-FOG, AI-Driven Freezing of Gait; FOG, freezing of gait; IMU, inertial measurement unit; %TF, percentage of time spent frozen; #FOG, number of FOG episodes.

Supplementary Video 1 Live demonstration of the AID-FOG platform. The video shows a walkthrough of upload, model prediction display, annotation adjustment, and download workflow on the AID-FOG web application. Abbreviations: AID-FOG, AI-Driven Freezing of Gait.

Usability of the AID-FOG platform

The CSUQ results for each version of the AID-FOG platform and for ELAN are summarized in Supplementary Table 8. Across all subscales: system usefulness, information quality, interface quality, and overall usability, the scores of the platform steadily increased, indicating that our expert raters found each version of the platform during each iteration more usable than the last. By the final version, AID-FOG's usability ratings had surpassed those of ELAN, although this comparison contains bias and was not conducted under controlled conditions. In future work, we will carry out a systematic evaluation of the usability and efficiency of the platform against established tools.

The benefits of the AID-FOG platform compared to ELAN were also highlighted in the interviews. For instance, when discussing improvements for Version 1.0 over ELAN, the convenience of annotation was frequently mentioned. Clinicians emphasized that the processes of adjusting, creating, and deleting episodes should be seamless and at least as efficient as in ELAN. Clinician A explained, "The start and end time of what the algorithm provides should be adjustable and very user-friendly." Clinician B added, "I think the core is that you have a very user-friendly way to adjust the freezing annotations currently, and I believe that should be the top priority at the moment." Moreover, when comparing Version 2.1 with ELAN, clinicians noted that AID-FOG integrated all ELAN functionalities and offered additional features like shortcuts and statistics. Clinician C commented, "I see potential in it, and once you get used to this system, you can become very efficient with it." Also, all clinicians expressed a positive view on switching from ELAN to the AID-FOG platform. They believed they could make better and faster progress with AID-FOG. Clinician C remarked, "As it is now, it's incredibly good. I'm really impressed. It's really chic."

References

[1] Yi, F., Wen, H. & Jiang, T. *Asformer: Transformer for action segmentation* (2021). URL https://api.semanticscholar.org/CorpusID:239016587.

[2] O'Day, J. *et al.* Assessing inertial measurement unit locations for freezing of gait detection and patient preference. *J. Neuroeng. Rehabil.* **19**, 20 (2022).

[3] Salomon, A. *et al.* A machine learning contest enhances automated freezing of gait detection and reveals time-of-day effects. *Nature Communications* **15**, 4853 (2024).

[4] Zhang, W. *et al.* Multimodal data for the detection of freezing of gait in parkinson's disease. *Scientific data* **9**, 606 (2022).

Type	#	Question	AID-FOG Platform			ELAN
			Version 1.0	Version 2.1	Version 2.2	
**System Usefulness (SYSUSE)**						
	1	Overall, I am satisfied with how easy it is to use this system	4.7	5.3	6.3	5.0
	2	It was simple to use this system	4.0	5.0	5.3	5.0
	3	I can effectively complete my work using this system	4.3	4.7	6.0	5.7
	4	I am able to complete my work quickly using this system	3.7	5.7	6.0	3.0
	5	I am able to efficiently complete my work using this system	3.3	5.0	6.0	3.3
	6	I feel comfortable using this system	5.3	5.0	6.3	5.3
	7	It was easy to learn to use this system	5.3	4.7	6.0	4.7
	8	I believe I became productive quickly using this system	3.7	5.3	6.3	5.0
**Information Quality (INFOQUAL)**						
	9	The system gives error messages that clearly tell me how to fix problems	2.0	5.0	5.3	2.7
	10	Whenever I make a mistake using the system, I recover easily and quickly	3.3	4.0	7.0	5.7
	11	The information (help/messages/documentation) provided is clear	3.3	5.0	5.3	3.3
	12	It is easy to find the information I needed	3.3	4.7	6.3	2.7
	13	The information provided is easy to understand	5.3	4.3	6.3	3.3
	14	The information is effective in helping me complete tasks	4.3	5.0	6.3	4.3
	15	The organization of information on the screens is clear	5.0	5.0	6.7	4.7
**Interface Quality (INTERQUAL)**						
	16	The interface of this system is pleasant	4.7	6.0	6.7	3.3
	17	I like using the interface of this system	4.7	5.3	6.3	3.3
	18	This system has all the functions and capabilities I expect	4.7	4.7	7.0	4.7
**Overall Usability**						
	19	Overall, I am satisfied with this system	5.0	5.7	7.0	4.3

Supplementary Table 8 CSUQ Scores for Different Versions of the AID-FOG platform and ELAN

[5] Mancini, M. *et al.* Technology for measuring freezing of gait: Current state of the art and recommendations. *Journal of Parkinson's Disease* 1877718X241301065 (2025).

[6] Mancini, M. *et al.* Measuring freezing of gait during daily-life: an open-source, wearable sensors approach. *J. Neuroeng. Rehabil.* **18**, 1 (2021).

[7] Lewis, J. R. Ibm computer usability satisfaction questionnaires: psychometric evaluation and instructions for use. *International Journal of Human-Computer Interaction* **7**, 57–78 (1995).

[8] Nijmegen: Max Planck Institute for Psycholinguistics, T. L. A. Elan (2024). URL https://archive.mpi.nl/tla/elan.

[9] Gilat, M. How to annotate freezing of gait from video: A standardized method using Open-Source software. *J. Parkinsons. Dis.* **9**, 821–824 (2019).
